# Neonatal Diet Impacts Circulatory miRNA Profile in a Porcine Model

**DOI:** 10.3389/fimmu.2020.01240

**Published:** 2020-06-23

**Authors:** Laura E. Carr, Anne K. Bowlin, Ahmed A. Elolimy, Stephanie D. Byrum, Charity L. Washam, Christopher E. Randolph, Stewart L. MacLeod, Laxmi Yeruva

**Affiliations:** ^1^Arkansas Children's Nutrition Center, Little Rock, AR, United States; ^2^Department of Pediatrics, University of Arkansas for Medical Sciences, Little Rock, AR, United States; ^3^Department of Biochemistry and Molecular Biology, University of Arkansas for Medical Sciences, Little Rock, AR, United States; ^4^Arkansas Children's Research Institute, Little Rock, AR, United States

**Keywords:** breastmilk, infant formula, miRNA, piglet, blood

## Abstract

microRNAs (miRNAs) are conserved non-coding small nucleotide molecules found in nearly all species and breastmilk. miRNAs present in breastmilk are very stable to freeze-thaw, RNase treatment, and low pH as they are protected inside exosomes. They are involved in regulating several physiologic and pathologic processes, including immunologic pathways, and we have demonstrated better immune response to vaccines in piglets fed with human milk (HM) in comparison to dairy-based formula (MF). To understand if neonatal diet impacts circulatory miRNA expression, serum miRNA expression was evaluated in piglets fed HM or MF while on their neonatal diet at postnatal day (PND) 21 and post-weaning to solid diet at PND 35 and 51. MF fed piglets showed increased expression of 14 miRNAs and decreased expression of 10 miRNAs, relative to HM fed piglets at PND 21. At PND 35, 9 miRNAs were downregulated in the MF compared to the HM group. At PND 51, 10 miRNAs were decreased and 17 were increased in the MF relative to HM suggesting the persistent effect of neonatal diet. miR-148 and miR-181 were decreased in MF compared to HM at PND 21. Let-7 was decreased at PND 35 while miR-199a and miR-199b were increased at PND 51 in MF compared to HM. Pathway analysis suggested that many of the miRNAs are involved in immune function. In conclusion, we observed differential expression of blood miRNAs at both PND 21 and PND 51. miRNA found in breastmilk were decreased in the serum of the MF group, suggesting that diet impacts circulating miRNA profiles at PND 21. The miRNAs continue to be altered at PND 51 suggesting a persistent effect of the neonatal diet. The sources of miRNAs in circulation need to be evaluated, as the piglets were fed the same solid diet leading up to PND 51 collections. In conclusion, the HM diet appears to have an immediate and persistent effect on the miRNA profile and likely regulates the pathways that impact the immune system and pose benefits to breastfed infants.

## Introduction

The World Health Organization and American Academy of Pediatrics recommend exclusive breastfeeding for the first six months of life, followed by breastfeeding with complimentary foods until 1 year of age (1, 2). It is well-established that breastfed babies have decreased rates of obesity, infections such as otitis media and respiratory tract infections, and decreased asthma and atopic dermatitis (2). However, the exact mechanisms that make breastfeeding better for infants is still unclear. Multiple components of breastmilk have been shown to impact growth and development as well as immune function including human milk oligosaccharides (3–5), immunoglobulins (6), cytokines (7, 8), and growth factors (9, 10). microRNA (miRNA) are also possible contributors to the benefits of breastfeeding.

miRNAs are conserved non-coding small nucleotide (~22 nucleotides) molecules (11) that have biological activities in humans (12–15). Breastmilk miRNAs are thought to survive in an acidic environment in the gastrointestinal tract, when exposed to RNase, and be absorbed in the gut (16). miRNAs from bovine milk have been found in the plasma of humans and noted to have a regulatory effect on cell functions (14), such as innate immune, T-cell and B-cell function; several of these miRNAs are also highly abundant in human milk (16, 17). Infant formulas, however, have a decreased amount of miRNA (13, 18). Dietary sources have been shown to contribute an appreciable amount of miRNA to total serum miRNA. For example, when mice are fed a miRNA-depleted cow's milk diet for 4 weeks, they showed a decrease in measured plasma miRNA by ~ 60% compared to mice fed a miRNA-sufficient diet (19). However, studies are limited in terms of understanding the impact of breastmilk miRNAs and other components on infants' health. Therefore, the purpose of the current study is to determine if neonatal diet influences serum miRNA and if it continues to have an impact after being weaned to a solid diet.

## Materials and Methods

### Animal Study

The piglet study design has been described previously (20). Animal maintenance and experimental protocols followed the ethical guidelines for animal research approved by the Institutional Animal Care and Use Committee (IACUC) and Institutional Biosafety Committee (IBC) at University of Arkansas for Medical Sciences. Briefly, 2 day old male piglets were obtained from a regional commercial farm and transferred to the vivarium at Arkansas Children's Nutrition Center (ACNC). They were then randomized to be fed an isocaloric diet of either dairy-based formula (MF; *n* = 26) or human breastmilk (HM; *n* = 26). Donor human breastmilk was obtained from the Mother's Milk Bank of North Texas, and Similac Advance powder was obtained from Ross Products (Abbot Laboratories). Both HM and MF diets were supplemented to meet the nutritional recommendations of the National Research Council (NRC) for growing piglets. At postnatal day (PND) 14, solid pig starter was introduced until PND 21, at which time all piglets were weaned to an *ad libitum* solid diet until PND 51.

### Sample Processing

At 8 h of fasting, blood was collected on the morning of PND 21, 35, and 51 into PAXgene (Qiagen) Blood RNA Tubes. At PND 21 there were 9 MF and 9 HM, 4 MF and 4 HM at PND 35, and 9 MF and 10 HM at PND 51. Tubes were allowed to sit for 2 h at room temperature and then stored at −80°C. Prior to processing, the PAXgene tubes were moved from the −80°C to 4°C overnight and then allowed to sit at room temperature for 2 h. The PAXgene tubes were then centrifuged at 3000 × g using a swing-out rotor (Eppendorf 5810R Centrifuge) for 10 min, and samples were processed with the PAXgene Blood miRNA Kit (PreAnalytiX, Switzerland) to isolate blood RNA according to the commercial protocol. RNA samples were stored at −80°C until needed for small RNA library preparation.

A cDNA sequencing library for miRNA (miRs) was generated using standard methods of the QIAseq miRNA Library Kit (Qiagen, Germany). Small RNA sequencing libraries were constructed using Qiagen's QIAseq® miRNA Library Kit (96) (Qiagen, Germany, cat. 331502) according to the manufacturer's protocol. Briefly, adapter sequences were sequentially ligated to the 3′ and 5′ ends of miRNA in each sample. Adapter ligated miRNAs were then assigned unique molecular indexes (UMI) and simultaneously transcribed into single-stranded cDNA. This was followed by cDNA cleanup per the manufacturer's instruction, and construction of PCR-amplified Illumina compatible sequencing libraries, which involved ligating a 3′ sequencing adapter, and 1 of 48 indexed adapters (QIAseq miRNA NGS 96 index IL) during the amplification process. The sequencing libraries were then subjected to a second library cleanup and validated for fragment size and quantity using an Advanced Analytical Fragment Analyzer (AATI) and Qubit fluorometer (Life Technologies), respectively. Equal amounts of each library were then pooled and sequenced on a NextSeq 500 platform using high output flow cells to generate a ~5–10 million 75-base single end reads per sample (1 × 75bp SE). All sequencing was performed by the Center for Translational Pediatric Research (CTPR) Genomics Core at Arkansas Children's Research Institute (Little Rock, AR, USA).

### Statistical Analysis

Following demultiplexing, miRNA reads were quality checked using FastQC (https://www.bioinformatics.babraham.ac.uk/projects/fastqc/) and MultiQC (21). The fastq files that passed quality control were then adapter trimmed. miRNAs were quantified using Qiagen's primary QIAseq miRNA quantification tool available through GeneGlobe's data analysis center (https://geneglobe.qiagen.com/us/analyze/) against all organisms in miRBase (miRBase v21). miRNA's with low UMI-counts were then removed before downstream analysis. To retain the maximum number of interesting features, miRNA with a minimum of 10 counts-per-million (CPM) in at least 17 libraries were retained for further investigation. The filtered dataset was then normalized for compositional bias using trimmed mean of M values (TMM) (22, 23). edgeR's quasi-likelihood method (*glmQLFTest*) was used to identify differentially expressed miRNA between experimental groups (24–26).

### Pathway Analysis

The challenge associated with the piglet model includes finding databases that support miRNA target prediction analysis. As miRNAs are conserved (27–29) and the pig genome is not well-annotated, a human miRNA database was utilized to conduct target prediction analysis using Ingenuity Pathway Analysis software (IPA, Qiagen). The experimentally verified target gene list of miRNA was generated. The target genes were subjected to canonical pathway analysis that included metabolic pathways, cell cycle regulation, cell growth, proliferation and development, cellular immune response, cellular stress and injury, cytokine signaling, growth factor signaling, humoral immune response, nuclear receptor signaling, organismal growth and development, pathogen-influenced signaling, and transcriptional regulation. The enriched pathways were based on the right-tailed Fisher's exact test (adjusted for False Discover Rate at 5%) that are graphed as negative log *p*-value. These pathways indicate the likelihood of an association of genes to the pathway in MF vs. HM fed piglets at different time points.

## Results

### miRNA Expression Profile

miRNA expression analysis was performed on blood samples from MF piglets in comparison to HM piglets at different time points (PND 21, 35, and 51). The reader is referred to Tables S1–S3 for miRNAs identified using human, mouse, and piglet genome. The data described here are exclusively based on piglet genome. Results demonstrate differential expression of miRNA in the MF group relative to HM fed piglets. At PND 21, 10 miRs were downregulated and 14 were upregulated in MF in comparison to HM fed piglets (Table 1). At PND 35, 9 miRs were decreased in MF relative to HM fed piglets (Table 2). At PND 51, 10 miRs were downregulated and 17 were upregulated in MF compared to HM fed piglets (Table 3). There were several miRNAs that displayed altered directionality depending on PND. For instance, ssc-miR-18b was increased in MF at PND 21 and decreased at PND 35 and PND 51 relative to HM group. ssc-miR-126-3p was elevated in MF compared to HM group at PND 21 and lower at PND 35. Other miRNAs were different only at certain time points. For example, ssc-miR-708-5p was decreased at PND 21 and PND 51 in the MF group relative to the HM group. ssc-miR-135 and ssc-miR-27b-3p were lower at both PND 35 and PND 51 in MF compared to HM group. In addition, miRs found in breastmilk by other research groups (13, 14, 16) such as miR-148 and miR-181 were decreased in MF compared to HM at PND 21. Furthermore, immune system related miRs such as let-7 (30–33) was decreased at PND 35 while miR-199a and miR-199b (34–36) were increased at PND 51 in MF compared to HM.

**Table 1:** MF fed piglets have differential miRNA expression at PND 21 relative to HM fed piglets.

**miRNA**	**FC**	***p*-value**
ssc-miR-708-5p	−39.76665575	0.002
ssc-miR-196b-5p	−3.237684325	0.001
ssc-miR-142-3p	−2.797240418	0.005
ssc-miR-7142-3p	−2.661387334	0.007
ssc-miR-181b	−2.420461905	0.006
ssc-miR-181d-5p	−2.28856399	0.018
ssc-miR-451	−2.276169814	0.012
ssc-miR-181a	−1.857234399	0.038
ssc-miR-1296-5p	−1.49268688	0.019
ssc-miR-148b-3p	−1.359814304	0.045
ssc-miR-28-3p	1.509698075	0.041
ssc-miR-532-5p	1.534975917	0.026
ssc-miR-128	1.559663023	0.019
ssc-miR-574	1.589728656	0.042
ssc-miR-9810-3p	1.613911517	0.030
ssc-miR-335	1.791816536	0.011
ssc-miR-1468	1.796667674	0.048
ssc-miR-7	1.809841726	0.023
ssc-miR-182	1.825348966	0.043
ssc-miR-126-3p	1.928895853	0.022
ssc-miR-99b	1.954175359	0.007
ssc-miR-130a	2.463583469	0.046
ssc-miR-142-5p	2.576233506	0.010
ssc-miR-18b	37.26653433	0.010

*Negative fold change (FC) indicates the miRNA is downregulated in MF fed piglets compared to HM fed piglets while positive FC indicates miRNA are upregulated in MF relative to HM group. The data represents values for 9 MF and 9 HM*.

**Table 2:** MF fed piglets have differential miRNA expression at PND 35 relative to HM fed piglets.

**miRNA**	**FC**	***p*-value**
ssc-miR-18b	−49.72067945	0.048
ssc-miR-135	−42.25878671	0.040
ssc-miR-9	−34.874668	0.049
ssc-miR-32	−7.975548269	0.047
ssc-miR-126-5p	−5.646933269	0.012
ssc-miR-27b-3p	−3.406773728	0.040
ssc-miR-126-3p	−2.826728093	0.051
ssc-miR-628	−2.647555179	0.053
ssc-let-7g	−1.964276243	0.012

*Negative fold change (FC) indicates the miRNA is downregulated in MF fed piglets compared to HM fed piglets. The data represents values for 4 MF and 4 HM*.

**Table 3:** MF fed piglets have differential miRNA expression at PND 51 relative to HM fed piglets.

**miRNA**	**FC**	***p*-value**
ssc-miR-708-5p	−181.4471982	0.000
ssc-miR-18b	−24.60941262	0.005
ssc-miR-135	−9.524924918	0.003
ssc-miR-23b	−2.890749819	0.012
ssc-miR-27b-3p	−2.835459396	0.005
ssc-miR-27a	−2.012577166	0.009
ssc-miR-28-5p	−1.795200855	0.040
ssc-miR-24-3p	−1.769462259	0.011
ssc-miR-99a	−1.55286892	0.046
ssc-miR-23a	−1.464557853	0.024
ssc-miR-339	1.393039587	0.040
ssc-miR-339-3p	1.431395735	0.041
ssc-miR-339-5p	1.435181521	0.028
ssc-miR-4334-3p	1.495246365	0.019
ssc-miR-532-3p	1.529235062	0.032
ssc-miR-1307	1.534150221	0.032
ssc-miR-149	1.63922384	0.053
ssc-miR-328	1.745143555	0.044
ssc-miR-320	1.872187384	0.016
ssc-miR-30c-3p	1.901539247	0.029
ssc-miR-199a-3p	2.200123182	0.006
ssc-miR-199b-3p	2.857399975	0.022
ssc-miR-100	3.033459346	0.029
ssc-miR-7139-3p	3.289366861	0.019
ssc-miR-199a-5p	3.552896912	0.000
ssc-miR-204	5.124808887	0.045
ssc-miR-205	8.226824537	0.002

*Negative fold change (FC) indicates the miRNA is downregulated in MF fed piglets compared to HM fed piglets while positive FC indicates miRNA are upregulated in MF relative to HM group. The data represents 9 MF and 10 HM*.

### Target Gene Prediction and Pathway Analysis of miRNAs

**Figure 1:**
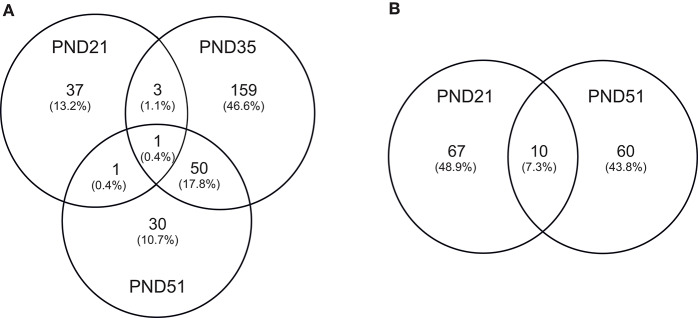
Target predicted genes in MF fed piglets relative to HM fed piglets at PND 21, 35 and 51. **(A)** Venn diagram shows unique and shared genes of downregulated miRNA in MF relative to HM at each time point. **(B)** Venn diagram shows unique and shared genes of upregulated miRNA in MF relative to HM at each time point. The data represents from piglets of 9 MF and 9 HM at PND21, 4 MF and 4 HM at PND 35, and 9 MF and 10 HM at PND 51.

**Figure 2:**
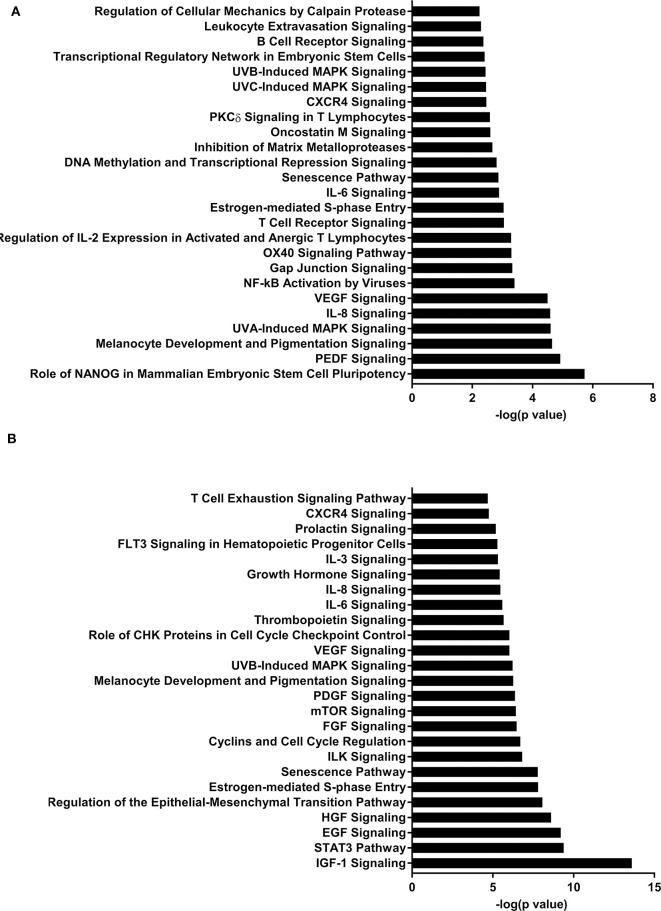
Enriched pathways of MF compared to HM at PND21. **(A)** IPA of target predicted genes for downregulated miRNA in MF relative to HM at PND21. **(B)** IPA of target predicted genes for upregulated miRNA in MF relative to HM at PND21. The data represents from 9 MF and 9 HM fed piglets.

**Figure 3:**
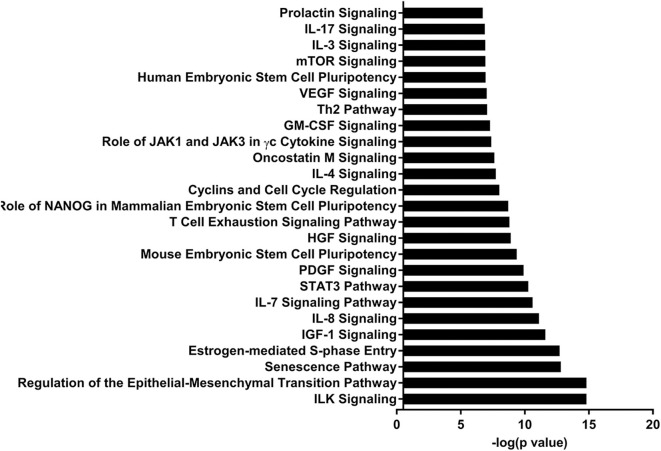
Enriched pathways of MF compared to HM at PND35. IPA of target predicted genes for downregulated miRNA in MF relative to HM at PND35. The data represents from 4 MF and 4 HM fed piglets.

**Figure 4:**
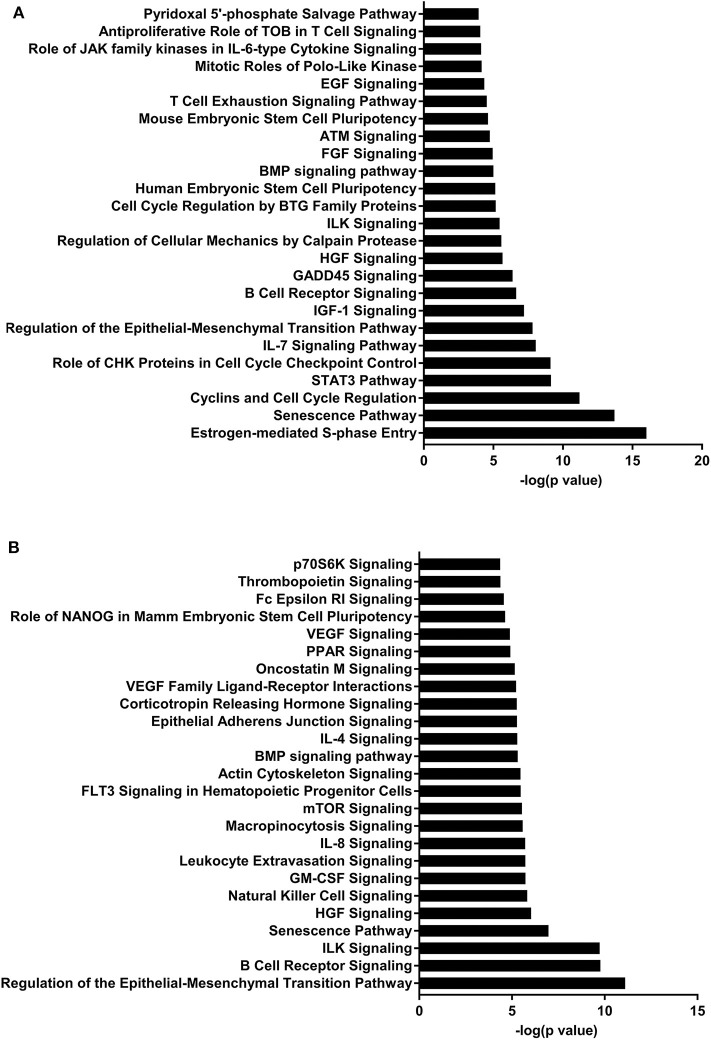
Enriched pathways of MF compared to HM at PND51. **(A)** IPA of target predicted genes for downregulated miRNA in MF relative to HM at PND51. **(B)** IPA of target predicted genes for upregulated miRNA in MF relative to HM at PND51. The data represents from 9 MF and 10 HM fed piglets.

IPA identified 17 (out of 24) miRs at PND 21, 7 (out of 9) at PND 35, and 15 (out of 27) at PND 51 with experimentally validated gene targets. miRNAs repress gene translation, therefore, downregulated miRNA is associated with increased gene expression and upregulated miRNA is associated with the decreased gene expression. For downregulated miRNAs in the MF group vs. HM group, the number of unique genes were 37 at PND 21, 159 at PND 35, and 30 at PND 51 (Figure 1A). The three common genes between PND 21 and PND 35 are B-cell lymphoma 2 like 1 (*BCL2L1*), Kristen rat sarcoma viral oncogene homolog (*KRAS*), and Vinisin-like 1 (*VSNL1*). The common genes between PND 21 and PND 51 is mitotic arrest deficient 2 like 1 (*MAD2L1*). There are 50 common genes between PND 35 and PND 51. There is one common gene, estrogen receptor 1 (*ESR1*), between PND 21, PND 35, and PND 51. For upregulated miRNA, unique genes were 67 at PND 21 and 60 at PND 51 (Figure 1B). There are ten common genes between PND 21 and PND 51. Pathway analysis of the target-predicted genes was performed using IPA in order to further understand the functions possibly regulated in the MF vs. HM group. The top 25 pathways are shown for the different time points in Figures 2– 4. A full list of genes and pathways possibly regulated by miRNA can be found in Tables S4–S8.

## Discussion

Breastfed infants, compared to formula fed, have decreased rates of infections such as otitis media, respiratory tract infections, gastroenteritis, and necrotizing enterocolitis as well as lower rates of obesity and diabetes (2). In previously published work (20), our lab noted that the piglets fed HM had higher serum antibody titers to cholera toxin subunit B and tetanus toxoid than those fed MF. They also had elevated immunoglobulin A producing cells specific to cholera toxin subunit B. The HM fed piglets were noted to have higher T cell proliferation compared to the MF group. There was no difference in body weights or caloric intake between the two groups, thus differences attributed here are likely by diet. Many components of breastmilk contribute to these improved outcomes in infants and new literature suggests miRNA may play a role. While there are studies that describe the different types of miRNA in breastmilk (13, 14, 16), there is no concrete evidence that miRNAs have a direct impact on infant immunity. It is also possible that other breastmilk components alone impact infant circulatory miRNA. To address this, we used a model of formula vs. breastmilk fed piglets collecting circulatory miRNA at different time points of weaning and post-weaning of the neonatal diet.

Kosaka et al. (16) noted expression of multiple miRNA in breastmilk that were predicted to be involved in T- and B-cell function. Specifically, miR-181a and miR-181b were identified in breastmilk. Interestingly, these miRs were decreased in circulation at PND 21 in our MF group compared to the HM group, suggesting that breastmilk could be the source for these miRNAs. Since these miRNAs are thought to be involved in B- and T-cell differentiation (37), it is plausible that the higher expression in HM fed piglets contributed to the diet-dependent differences in immune cell activity that we previously reported in these animals. The expression pattern of miR-181a during T-cell maturation is dynamic and likely influences development of T-cells (38). miR-181 also plays a role in inflammation. It has been shown to downregulate production of TNF-α in *Brucella abortus* infections (39). These data, along with ours, suggest that breastmilk miRs are likely involved in protecting infants in modulating the immune system (i.e., to reduce inflammation by infection and to impact T-cell maturation).

Golan-Gerstl et al. and Kahn et al. both showed high levels of miR-148 in pre-term, early term, and term breastmilk (13, 40). Golan-Gerstl et al. also showed significantly reduced amounts of miR-148 in formula compared to breastmilk. The piglets fed MF had a decreased amount of blood miR-148 compared to those fed HM at PND 21. miR-148 family negatively regulates the innate immune response by limiting cytokine production and inhibiting T-cell proliferation initiated by dendritic cell presentation of antigens in a mouse model (41), suggesting a role in reducing inflammatory cytokine production in HM fed piglets.

Let-7 is highly present in both the skim and fat layers of breastmilk (13, 14, 42). It has also been shown in these layers in bovine and goat milk (13). Let-7 regulates the innate and adaptive immune response, plays a role in TLR4 signaling and macrophage activity, and also affects T-cell differentiation and limits B-cell activation (30–33). At PND 35, let-7 had decreased concentration in MF fed group compared to HM group.

At both PND 21 and PND 51, miR-708-5p was significantly decreased in the MF fed piglets compared to the HM fed piglets, ~40 fold and 180 fold respectively (Tables 1, 3). miR-708 has been shown to target TLR4 (34) suggesting decreased inflammatory pathway activation in HM fed piglets compared to MF fed. miR-708 has also been shown to increase phagocytosis (35) which may allow the HM fed piglets to eliminate pathogens more easily than the MF fed piglets. miR-18b was significantly upregulated at PND 21 but significantly downregulated at PND 35 and 51 in the MF compared to HM fed piglets (Tables 1–3). In patients with multiple sclerosis, miR-18b has been associated with relapse (36, 43) so it is possible that it plays a role in inflammation and autoimmune diseases.

The piglets fed MF had higher levels of miR-199a and miR-199b at PND 51 than those fed HM. miR-199b has been found to be significantly increased in nasal mucous extracellular vesicles of adults with allergic rhinitis compared to those that are healthy (44). In asthma patients with a neutrophilic phenotype, plasma miR-199a was significantly increased and correlated negatively with pulmonary function (45). Wang et al. showed in a mouse model infected with *Mycobacterium bovis* that miRNA-199a inhibits autophagy of macrophages and decreases interferon-β production. This allows *M. bovis* to survive and grow in these infected mice (46). miR-199 is associated with allergy and asthma in adults and with bacterial survival in mice. It is possible that this miRNA is involved in increased atopy in formula fed infants (2). These data suggest that the diet could have a persistent effect on miRNA expression and on the immune system.

Several studies have shown that diet alone impacts miRNA levels. In a review by Kura et al. (47), different dietary components such as vitamin D, selenium, and vitamin E impacted blood and cardiac miRNAs that are associated with decreased cardiovascular disease. A high fat diet is associated with decreased miRNA-29b expression in the heart and increases susceptibility to heart injury (48). Dietary compounds have also been shown to change the miRNA expression in skin in patients with psoriasis, helping with treatment of this disease (49). These data suggest that neonatal diet itself can impact miRNA expression. miRNA expression may have an impact in microbiome as well. Zhou et al. showed that mice fed an exosome/RNA depleted diet had different microbiome than mice fed an exosome/RNA sufficient diet (50). In our piglets, miRNA profiles are different in the formula fed vs. breastmilk fed piglets, as are the microbiome profiles [previously published data (51)]. While speculative, it is possible that the miRNA played a part in the neonatal diet-associated differences of the microbiome.

Pathway analysis revealed several pathways involved in immune function. B-cell receptor signaling pathway was likely upregulated in the MF compared to HM at PND 21 in both the blood and ileal mucosa (Elolimy et al., unpublished results). The B-cell receptor pathway helps with development and differentiation of B-cells after exposure to antigens (52, 53). HM contains immunoglobulins (6, 54) that help in gut mucosa development and likely immune system education. These are not present in formula, which is likely the reason for an upregulation in this signaling pathway in the formula group. In the blood, this pathway is also both increased and decreased at PND 51, which is possibly due to the fact that signaling by miRNA is involved in maintaining homeostasis in the host. DNA methylation and transcriptional repression signaling pathway was also increased in the MF vs. HM group at PND 21 in both the blood and the ileal mucosa (Elolimy et al., unpublished results). DNA methylation involves regulation of gene expression by either inhibiting binding of transcription factor(s) or recruiting gene repression proteins to bind the DNA (55). This implies that MF fed may have a different methylation pattern and therefore gene expression, than HM fed, possibly these impact immune and metabolic realms.

The IL-7 signaling pathway is likely upregulated at PND 35 and 51 in the MF group compared to the HM group (miRNA were decreased). The IL-7 signaling pathway is important for development and differentiation of T-cells and early development of B-cells (56). Multiple cytokines have been found in human milk including IL-7 (57, 58) so it is possible that the HM group did not have an increase in this pathway because they are already exposed to IL-7 from the HM. This also suggests that the neonatal diet has prolonged effects on miRNA and gene expression post-weaning neonatal diet.

The IGF-1 signaling pathway was downregulated at PND 21 (miRNA upregulated) and upregulated at both PND 35 and 51 (miRNA downregulated) in the MF group compared to the HM group. Insulin-like growth factor (IGF)-1 plays an important role in multiple areas of development including cell proliferation and differentiation of tissues (59). Low levels of IGF-1 have been associated with different complications in premature infants including retinopathy of prematurity (ROP) (60) and bronchopulmonary dysplasia (BPD) (61). Interestingly, one study looked at IGF-1 to prevent these complications and decrease rates of ROP (60) which further prompted an ongoing study looking at IGF-1 infusion to prevent BPD. IGF-1 has also been shown in rat models to decrease germinal matrix hemorrhage bleeds (62).

There are several limitations to this study. First, the human breastmilk used was a pool and pasteurized. While miRNA has been shown to survive pasteurization (17), several other components might not survive the pasteurization process. Lactoferrin and secretory IgA are both reduced to some extent by pasteurization (63). We were not able to isolate miRNA from the breast milk samples at the time of this study, therefore, the differences seen in the MF vs. HM fed group are possibly attributable to human milk miRNA, but could also be due to other components in breast milk such as secretory IgA, human milk oligosaccharides, cytokines, etc (3–10). Secondly, the age of the babies of the donor milk mothers varies from about 2 months to 12 months (with an average of 6 months) and breastmilk components change over time (64, 65); thus, differences observed cannot be attributed to specific postpartum milk. Since the source of miRNA in this study was whole blood, future studies are needed to determine the specific cell types involved in miRNA expression profile.

## Conclusion

Human breastmilk fed piglets were found to have variable amounts of circulatory miRNA compared to formula fed piglets in our pilot study. We proposed that the differential abundances of miRNA impacts immune system in MF vs. HM fed piglets. Further studies should include a human study of serum miRNA in breastmilk vs. formula fed infants as well as miRNA present in their diets. Also, studies looking at specific immune cells and their roles/associations with the miRNA patterns are warranted.

## Data Availability Statement

The raw data supporting the conclusions of this article will be made available by the authors, without undue reservation, to any qualified researcher.

## Ethics Statement

The animal study was reviewed and approved by Institutional Animal Care and Use Committee at the University of Arkansas for Medical Sciences.

## Author Contributions

LC conducted the RNA isolation, library preparation of study samples, interpreted the data, wrote the manuscript and is responsible for the final content of the manuscript. AB conducted the piglet study. AE performed IPA analysis. SB and CW performed the miRNA sequencing data and statistical analysis. CR and SM performed sequencing. LY acquired the funding, designed the study, edited the manuscript and is responsible for the final content of the manuscript and the principal investigator of this study. All authors contributed to the article and approved the submitted version.

## Conflict of Interest

The authors declare that the research was conducted in the absence of any commercial or financial relationships that could be construed as a potential conflict of interest.
