# Supplementary material for: Neonatal Diet Impacts Circulatory miRNA Profile in a Porcine Model
Source: Front Immunol. 2020 Jun 23;11:1240. doi: 10.3389/fimmu.2020.01240 (PMC7324749; doi:10.3389/fimmu.2020.01240)
Supplement: Supplementary file 1 [file Table_1.DOCX]

**Table S1.** miRNA expression based on p-value at PND 21 using human, mouse, and/or piglet genome databases.

| **miRNA** | **Species** | **FC** | **p-Value** |
| --- | --- | --- | --- |
| hsa-miR-196b-5p, mmu-miR-196b-5p, ssc-miR-196b-5p | Human, mouse, pig | -3.378037353 | 0.000600255 |
| ssc-miR-142-3p | Pig | -3.012268861 | 0.003979628 |
| hsa-miR-181d-5p, mmu-miR-181d-5p | Human, mouse | -2.819775883 | 0.00272565 |
| ssc-miR-7142-3p | Pig | -2.724329476 | 0.006282116 |
| hsa-miR-142-3p, mdo-miR-142, mmu-miR-142a-3 | Human, mouse | -2.620187621 | 0.002648959 |
| mmu-miR-148b-5p | Mouse | -2.580005063 | 0.004033446 |
| ssc-miR-181b | Pig | -2.497899791 | 0.00469625 |
| hsa-miR-451a, mmu-miR-451a, ssc-miR-451 | Human, mouse, pig | -2.420657535 | 0.008874014 |
| hsa-miR-7977 | Human | -2.298203233 | 0.030483749 |
| hsa-miR-1271-3p | Human | -1.992098355 | 0.030806346 |
| hsa-miR-23b-3p, mmu-miR-23b-3p | Human, mouse | -1.959373827 | 0.053300225 |
| ssc-miR-181a | Pig | -1.940860271 | 0.024247765 |
| hsa-miR-5100 | Human | -1.887622963 | 0.017790731 |
| hsa-miR-181a-5p, mmu-miR-181a-5p | Human, mouse | -1.817132479 | 0.031701514 |
| hsa-miR-1303 | Human | -1.785770728 | 0.008041748 |
| hsa-miR-342-5p | Human | -1.755797702 | 0.004326095 |
| ssc-miR-20a | *Pig* | -1.738601563 | 0.036176349 |
| hsa-miR-181b-5p, mmu-miR-181b-5p | Human, mouse | -1.686895485 | 0.018469152 |
| hsa-miR-1296-5p, ssc-miR-1296-5p | Human, pig | -1.515603077 | 0.023464111 |
| hsa-miR-362-5p | Human | -1.440938887 | 0.034552191 |
| hsa-miR-148b-3p, mmu-miR-148b-3p, ssc-miR-148b-3p | Human, mouse, pig | -1.40205767 | 0.039360776 |
| hsa-miR-128-3p, mmu-miR-128-3p, ssc-miR-128 | Human, mouse, pig | 1.4142846 | 0.041162029 |
| hsa-miR-361-3p | Human | 1.427306877 | 0.049507488 |
| mmu-miR-1983 | mouse | 1.436509357 | 0.0333745 |
| hsa-miR-532-5p, mmu-miR-532-5p, ssc-miR-532-5p | Human, mouse, pig | 1.4410305 | 0.048574729 |
| mmu-miR-361-3p | Mouse | 1.467463066 | 0.045311667 |
| mmu-miR-8092 | Mouse | 1.49989851 | 0.048709358 |
| hsa-miR-335-5p, mmu-miR-335-5p | Human, mouse | 1.545338762 | 0.015452551 |
| ssc-miR-9810-3p | Pig | 1.551636761 | 0.03613302 |
| ssc-miR-7 | Pig | 1.621175158 | 0.045050578 |
| hsa-miR-3529-3p | Human | 1.637402456 | 0.025507137 |
| hsa-miR-125a-5p, mmu-miR-125a-5p | Human, mouse | 1.646273585 | 0.049336032 |
| ssc-miR-335 | Pig | 1.657220733 | 0.021692675 |
| hsa-miR-628-3p | Human | 1.671030238 | 0.015123893 |
| hsa-miR-126-3p, mmu-miR-126a-3p, ssc-miR-126-3p | Human, mouse, pig | 1.80203539 | 0.033563741 |
| hsa-miR-7-5p, mmu-miR-7a-5p | Human, mouse | 1.823297344 | 0.021142992 |
| hsa-miR-99b-5p, mmu-miR-99b-5p, ssc-miR-99b | Human, mouse, pig | 1.934716087 | 0.008394057 |
| hsa-miR-142-5p, mmu-miR-142a-5p, ssc-miR-142-5p | Human, mouse, pig | 2.2540734 | 0.022339725 |
| mmu-miR-3969 | Mouse | 2.572052336 | 0.001054132 |
| mmu-miR-6928-5p | Mouse | 2.720148277 | 0.046324052 |
| hsa-miR-3168 | Human | 4.396956233 | 0.038803817 |
| hsa-miR-5694 | Human | 5.013958541 | 0.048973555 |

Negative fold change (FC) indicates the miRNA is downregulated in MF fed piglets compared to HM fed piglets and positive FC indicates that miRNA is upregulated in MF fed piglets relative to HM fed piglets.
