# Supplementary material for: Neonatal Diet Impacts Circulatory miRNA Profile in a Porcine Model
Source: Front Immunol. 2020 Jun 23;11:1240. doi: 10.3389/fimmu.2020.01240 (PMC7324749; doi:10.3389/fimmu.2020.01240)
Supplement: Supplementary file 4 [file Table_4.DOCX]

**Table S4. List of genes and enriched pathways of downregulated miRNA in MF compared to HM group at PND 21.**

| **Canonical Pathways** | **-log(p-value)** | **Genes** |
| --- | --- | --- |
| Role of NANOG in Mammalian Embryonic Stem Cell Pluripotency | 5.73 | CDX2, GATA6, KRAS, LIFR, TCL1A |
| PEDF Signaling | 4.92 | BCL2, BCL2L1, IKBKB, KRAS |
| Melanocyte Development and Pigmentation Signaling | 4.65 | ADCY9, BCL2, KRAS, RPS6KA5 |
| UVA-Induced MAPK Signaling | 4.6 | BCL2L1, KRAS, PRKCA, RPS6KA5 |
| IL-8 Signaling | 4.59 | BCL2, BCL2L1, IKBKB, KRAS, PRKCA |
| VEGF Signaling | 4.5 | BCL2, BCL2L1, KRAS, PRKCA |
| NF-κB Activation by Viruses | 3.4 | IKBKB, KRAS, PRKCA |
| Gap Junction Signaling | 3.33 | ADCY9, GRIA2, KRAS, PRKCA |
| OX40 Signaling Pathway | 3.3 | BCL2, BCL2L1, TRA |
| Regulation of IL-2 Expression in Activated and Anergic T Lymphocytes | 3.29 | IKBKB, KRAS, TRA |
| T Cell Receptor Signaling | 3.05 | IKBKB, KRAS, TRA |
| Estrogen-mediated S-phase Entry | 3.04 | CDKN1B, ESR1 |
| IL-6 Signaling | 2.89 | ABCB1, IKBKB, KRAS |
| Senescence Pathway | 2.87 | CDKN1B, IKBKB, KRAS, RPS6KA5 |
| DNA Methylation and Transcriptional Repression Signaling | 2.81 | DNMT1, DNMT3B |
| Inhibition of Matrix Metalloproteases | 2.67 | MMP14, TIMP3 |
| Oncostatin M Signaling | 2.6 | KRAS, TIMP3 |
| PKCθ Signaling in T Lymphocytes | 2.59 | IKBKB, KRAS, TRA |
| CXCR4 Signaling | 2.47 | ADCY9, KRAS, PRKCA |
| UVC-Induced MAPK Signaling | 2.46 | KRAS, PRKCA |
| UVB-Induced MAPK Signaling | 2.44 | PRKCA, RPS6KA5 |
| Transcriptional Regulatory Network in Embryonic Stem Cells | 2.41 | CDX2, GATA6 |
| B Cell Receptor Signaling | 2.37 | BCL2L1, IKBKB, KRAS |
| Leukocyte Extravasation Signaling | 2.29 | MMP14, PRKCA, TIMP3 |
| Regulation of Cellular Mechanics by Calpain Protease | 2.24 | CDKN1B, KRAS |
| Thrombopoietin Signaling | 2.23 | KRAS, PRKCA |
| mTOR Signaling | 2.22 | KRAS, PRKCA, RPS6KA5 |
| GM-CSF Signaling | 2.18 | BCL2L1, KRAS |
| Growth Hormone Signaling | 2.13 | PRKCA, RPS6KA5 |
| Macropinocytosis Signaling | 2.12 | KRAS, PRKCA |
| Erythropoietin Signaling | 2.09 | KRAS, PRKCA |
| IL-3 Signaling | 2.09 | KRAS, PRKCA |
| FLT3 Signaling in Hematopoietic Progenitor Cells | 2.08 | KRAS, RPS6KA5 |
| IL-7 Signaling Pathway | 2.08 | BCL2, CDKN1B |
| Antiproliferative Role of Somatostatin Receptor 2 | 2.07 | CDKN1B, KRAS |
| Prolactin Signaling | 2.04 | KRAS, PRKCA |
| Chemokine Signaling | 2.04 | KRAS, PRKCA |
| FGF Signaling | 2 | PRKCA, RPS6KA5 |
| VEGF Family Ligand-Receptor Interactions | 1.98 | KRAS, PRKCA |
| PDGF Signaling | 1.96 | KRAS, PRKCA |
| IL-1 Signaling | 1.94 | ADCY9, IKBKB |
| CCR5 Signaling in Macrophages | 1.9 | PRKCA, TRA |
| Mouse Embryonic Stem Cell Pluripotency | 1.87 | KRAS, LIFR |
| PPAR Signaling | 1.86 | IKBKB, KRAS |
| Virus Entry via Endocytic Pathways | 1.79 | KRAS, PRKCA |
| HGF Signaling | 1.79 | KRAS, PRKCA |
| iCOS-iCOSL Signaling in T Helper Cells | 1.77 | IKBKB, TRA |
| Th1 Pathway | 1.73 | NOTCH4, TRA |
| Fc Epsilon RI Signaling | 1.72 | KRAS, PRKCA |
| fMLP Signaling in Neutrophils | 1.72 | KRAS, PRKCA |
| Natural Killer Cell Signaling | 1.71 | KRAS, PRKCA |
| CD28 Signaling in T Helper Cells | 1.71 | IKBKB, TRA |
| Eumelanin Biosynthesis | 1.69 | MIF |
| CCR3 Signaling in Eosinophils | 1.68 | KRAS, PRKCA |
| p70S6K Signaling | 1.65 | KRAS, PRKCA |
| IL-12 Signaling and Production in Macrophages | 1.64 | IKBKB, PRKCA |
| STAT3 Pathway | 1.64 | BCL2, KRAS |
| Th2 Pathway | 1.64 | NOTCH4, TRA |
| Estrogen Receptor Signaling | 1.63 | ESR1, KRAS |
| PI3K Signaling in B Lymphocytes | 1.6 | IKBKB, KRAS |
| Epithelial Adherens Junction Signaling | 1.54 | KRAS, NOTCH4 |
| Corticotropin Releasing Hormone Signaling | 1.53 | ADCY9, PRKCA |
| Salvage Pathways of Pyrimidine Deoxyribonucleotides | 1.47 | AICDA |
| Th1 and Th2 Activation Pathway | 1.45 | NOTCH4, TRA |
| T Cell Exhaustion Signaling Pathway | 1.42 | KRAS, TRA |
| Dendritic Cell Maturation | 1.4 | IKBKB, TRA |
| Regulation of the Epithelial-Mesenchymal Transition Pathway | 1.37 | KRAS, NOTCH4 |
| Lipid Antigen Presentation by CD1 | 1.36 | TRA |
| Antiproliferative Role of TOB in T Cell Signaling | 1.36 | CDKN1B |
| Production of Nitric Oxide and Reactive Oxygen Species in Macrophages | 1.36 | IKBKB, PRKCA |
| 4-1BB Signaling in T Lymphocytes | 1.27 | IKBKB |
| IL-17A Signaling in Fibroblasts | 1.23 | IKBKB |
| MIF-mediated Glucocorticoid Regulation | 1.22 | MIF |
| Interferon Signaling | 1.22 | BCL2 |
| Role of PKR in Interferon Induction and Antiviral Response | 1.17 | IKBKB |
| B Cell Activating Factor Signaling | 1.17 | IKBKB |
| Mechanisms of Viral Exit from Host Cells | 1.17 | PRKCA |
| Role of RIG1-like Receptors in Antiviral Innate Immunity | 1.14 | IKBKB |
| MIF Regulation of Innate Immunity | 1.13 | MIF |
| Role of Oct4 in Mammalian Embryonic Stem Cell Pluripotency | 1.12 | CDX2 |
| iNOS Signaling | 1.1 | IKBKB |
| Hematopoiesis from Pluripotent Stem Cells | 1.09 | TRA |
| EGF Signaling | 1.02 | PRKCA |
| Activation of IRF by Cytosolic Pattern Recognition Receptors | 0.987 | IKBKB |
| IL-2 Signaling | 0.987 | KRAS |
| CD40 Signaling | 0.975 | IKBKB |
| Autophagy | 0.975 | BCL2 |
| IL-17A Signaling in Airway Cells | 0.971 | IKBKB |
| Role of JAK1 and JAK3 in γc Cytokine Signaling | 0.951 | KRAS |
| IL-10 Signaling | 0.928 | IKBKB |
| T Helper Cell Differentiation | 0.921 | TRA |
| Caveolar-mediated Endocytosis Signaling | 0.917 | PRKCA |
| FcγRIIB Signaling in B Lymphocytes | 0.896 | KRAS |
| IL-17 Signaling | 0.889 | KRAS |
| Cyclins and Cell Cycle Regulation | 0.886 | CDKN1B |
| BMP signaling pathway | 0.86 | KRAS |
| IL-4 Signaling | 0.857 | KRAS |
| CTLA4 Signaling in Cytotoxic T Lymphocytes | 0.848 | TRA |
| Crosstalk between Dendritic Cells and Natural Killer Cells | 0.842 | CD69 |
| Th17 Activation Pathway | 0.83 | TRA |
| Communication between Innate and Adaptive Immune Cells | 0.812 | TRA |
| Fcγ Receptor-mediated Phagocytosis in Macrophages and Monocytes | 0.801 | PRKCA |
| IGF-1 Signaling | 0.777 | KRAS |
| Paxillin Signaling | 0.77 | KRAS |
| Salvage Pathways of Pyrimidine Ribonucleotides | 0.75 | AICDA |
| Antioxidant Action of Vitamin C | 0.745 | IKBKB |
| GP6 Signaling Pathway | 0.714 | PRKCA |
| Phagosome Formation | 0.712 | PRKCA |
| Androgen Signaling | 0.672 | PRKCA |
| White Adipose Tissue Browning Pathway | 0.66 | ADCY9 |
| Role of Pattern Recognition Receptors in Recognition of Bacteria and Viruses | 0.633 | PRKCA |
| Relaxin Signaling | 0.618 | ADCY9 |
| HMGB1 Signaling | 0.606 | KRAS |
| Cdc42 Signaling | 0.597 | TRA |
| Germ Cell-Sertoli Cell Junction Signaling | 0.587 | KRAS |
| Granulocyte Adhesion and Diapedesis | 0.572 | MMP14 |
| ILK Signaling | 0.55 | RPS6KA5 |
| Sertoli Cell-Sertoli Cell Junction Signaling | 0.548 | KRAS |
| Agranulocyte Adhesion and Diapedesis | 0.548 | MMP14 |
| Actin Cytoskeleton Signaling | 0.496 | KRAS |
| Sperm Motility | 0.472 | PRKCA |

The enriched pathways were based on the right-tailed Fisher’s exact test (adjusted for False Discover Rate at 5%) that are graphed as negative log p value. These pathways indicate the likelihood of an association of genes to the pathway in MF versus HM fed piglets at different time points.
