# Supplementary material for: Neonatal Diet Impacts Circulatory miRNA Profile in a Porcine Model
Source: Front Immunol. 2020 Jun 23;11:1240. doi: 10.3389/fimmu.2020.01240 (PMC7324749; doi:10.3389/fimmu.2020.01240)
Supplement: Supplementary file 5 [file Table_5.DOCX]

**Table S5. List of genes and enriched pathways of upregulated miRNA in MF compared to HM group at PND 21.**

| **Canonical Pathways** | **-log(p-value)** | **Genes** |
| --- | --- | --- |
| IGF-1 Signaling | 13.6 | CCN2, FOS, IGF1R, IRS1, IRS2, PIK3R2, PTPN11, PXN, RAF1, RASA1, SRF |
| STAT3 Pathway | 9.39 | CDKN1A, EGFR, FGFR3, IGF1R, NTRK3, PIAS3, RAF1, TGFBR1, VEGFA |
| EGF Signaling | 9.2 | EGFR, FOS, MTOR, PIK3R2, RAF1, RASA1, SRF |
| HGF Signaling | 8.6 | CDKN1A, CRKL, FOS, PAK1, PIK3R2, PTPN11, PXN, RAF1 |
| Regulation of the Epithelial-Mesenchymal Transition Pathway | 8.07 | EGFR, FGF16, FGFR3, HIF1A, PIK3R2, PTPN11, RAF1, SMAD4, TGFBR1 |
| Estrogen-mediated S-phase Entry | 7.8 | CDKN1A, E2F1, E2F2, E2F3, ESR1 |
| Senescence Pathway | 7.79 | CDKN1A, E2F1, E2F2, E2F3, EIF4E, MTOR, PIK3R2, RAF1, SMAD4, TGFBR1 |
| ILK Signaling | 6.82 | FOS, HIF1A, IRS1, IRS2, MTOR, PIK3R2, PXN, VEGFA |
| Cyclins and Cell Cycle Regulation | 6.7 | CDKN1A, E2F1, E2F2, E2F3, RAF1, WEE1 |
| FGF Signaling | 6.48 | CRKL, FGF16, FGFR3, PIK3R2, PTPN11, RAF1 |
| mTOR Signaling | 6.43 | EIF4E, HIF1A, IRS1, MAPKAP1, MTOR, PIK3R2, RPTOR, VEGFA |
| PDGF Signaling | 6.37 | CRKL, FOS, PIK3R2, RAF1, RASA1, SRF |
| Melanocyte Development and Pigmentation Signaling | 6.26 | ADCY6, KIT, MITF, PIK3R2, PTPN11, RAF1 |
| UVB-Induced MAPK Signaling | 6.23 | EGFR, EIF4E, FOS, MTOR, PIK3R2 |
| VEGF Signaling | 6.03 | HIF1A, PIK3R2, PTPN11, PXN, RAF1, VEGFA |
| Role of CHK Proteins in Cell Cycle Checkpoint Control | 6.03 | CDKN1A, E2F1, E2F2, E2F3, PLK1 |
| Thrombopoietin Signaling | 5.67 | FOS, IRS2, PIK3R2, PTPN11, RAF1 |
| IL-6 Signaling | 5.59 | FOS, PIK3R2, PTPN11, RAF1, SRF, VEGFA |
| IL-8 Signaling | 5.47 | EGFR, FOS, MTOR, PIK3R2, RAF1, VCAM1, VEGFA |
| Growth Hormone Signaling | 5.43 | FOS, IGF1R, IRS1, PIK3R2, SRF |
| IL-3 Signaling | 5.32 | CRKL, FOS, PAK1, PIK3R2, RAF1 |
| FLT3 Signaling in Hematopoietic Progenitor Cells | 5.29 | EIF4E, MTOR, PIK3R2, PTPN11, RAF1 |
| Prolactin Signaling | 5.19 | FOS, IRS1, PIK3R2, PTPN11, RAF1 |
| CXCR4 Signaling | 4.75 | ADCY6, FOS, PAK1, PIK3R2, PXN, RAF1 |
| T Cell Exhaustion Signaling Pathway | 4.69 | FOS, MTOR, PIK3R2, PTPN11, TGFBR1, VEGFA |
| IL-2 Signaling | 4.35 | FOS, PIK3R2, PTPN11, RAF1 |
| Role of JAK1 and JAK3 in γc Cytokine Signaling | 4.19 | IRS1, IRS2, PIK3R2, PTPN11 |
| p70S6K Signaling | 4.19 | EGFR, IRS1, MTOR, PIK3R2, RAF1 |
| Actin Cytoskeleton Signaling | 4.17 | CRKL, FGF16, PAK1, PIK3R2, PXN, RAF1 |
| Human Embryonic Stem Cell Pluripotency | 4.12 | FGFR3, NTRK3, PIK3R2, SMAD4, TGFBR1 |
| PI3K Signaling in B Lymphocytes | 4.07 | FOS, IRS1, IRS2, PIK3R2, RAF1 |
| Role of JAK2 in Hormone-like Cytokine Signaling | 3.79 | IRS1, IRS2, PTPN11 |
| VEGF Family Ligand-Receptor Interactions | 3.74 | FOS, PIK3R2, RAF1, VEGFA |
| Regulation of IL-2 Expression in Activated and Anergic T Lymphocytes | 3.73 | FOS, RAF1, SMAD4, TGFBR1 |
| Cell Cycle Regulation by BTG Family Proteins | 3.68 | E2F1, E2F2, E2F3 |
| UVA-Induced MAPK Signaling | 3.59 | EGFR, FOS, MTOR, PIK3R2 |
| Mouse Embryonic Stem Cell Pluripotency | 3.52 | PIK3R2, PTPN11, RAF1, SMAD4 |
| Sumoylation Pathway | 3.43 | AR, FOS, SMAD4, SNCA |
| T Cell Receptor Signaling | 3.41 | FOS, PIK3R2, RAF1, RASA1 |
| Leukocyte Extravasation Signaling | 3.37 | CRKL, PIK3R2, PTPN11, PXN, VCAM1 |
| UVC-Induced MAPK Signaling | 3.27 | EGFR, FOS, RAF1 |
| Natural Killer Cell Signaling | 3.21 | PAK1, PIK3R2, PTPN11, RAF1 |
| CD28 Signaling in T Helper Cells | 3.2 | FOS, PAK1, PIK3R2, PTPN11 |
| Sperm Motility | 3.05 | EGFR, FGFR3, IGF1R, KIT, NTRK3 |
| White Adipose Tissue Browning Pathway | 2.98 | ADCY6, FGFR3, RARG, VEGFA |
| Corticotropin Releasing Hormone Signaling | 2.85 | ADCY6, FOS, RAF1, VEGFA |
| GM-CSF Signaling | 2.85 | PIK3R2, PTPN11, RAF1 |
| Relaxin Signaling | 2.8 | ADCY6, FOS, PIK3R2, VEGFA |
| Macropinocytosis Signaling | 2.76 | CSF1, PAK1, PIK3R2 |
| Cdc42 Signaling | 2.72 | FOS, PAK1, RAF1, RASA1 |
| Erythropoietin Signaling | 2.71 | FOS, PIK3R2, RAF1 |
| Antiproliferative Role of Somatostatin Receptor 2 | 2.68 | CDKN1A, PIK3R2, PTPN11 |
| Germ Cell-Sertoli Cell Junction Signaling | 2.67 | PAK1, PIK3R2, PXN, TGFBR1 |
| PEDF Signaling | 2.67 | PIK3R2, RAF1, SRF |
| IL-4 Signaling | 2.6 | IRS1, MTOR, PIK3R2 |
| IL-17A Signaling in Gastric Cells | 2.56 | EGFR, FOS |
| B Cell Receptor Signaling | 2.53 | MTOR, PIK3R2, PTPN11, RAF1 |
| Antiproliferative Role of TOB in T Cell Signaling | 2.53 | SMAD4, TGFBR1 |
| Clathrin-mediated Endocytosis Signaling | 2.48 | FGF16, LDLR, PIK3R2, VEGFA |
| Fcγ Receptor-mediated Phagocytosis in Macrophages and Monocytes | 2.42 | PAK1, PIK3R2, PXN |
| Gap Junction Signaling | 2.37 | ADCY6, EGFR, PIK3R2, RAF1 |
| Paxillin Signaling | 2.33 | PAK1, PIK3R2, PXN |
| Role of NANOG in Mammalian Embryonic Stem Cell Pluripotency | 2.21 | PIK3R2, RAF1, SMAD4 |
| Fc Epsilon RI Signaling | 2.17 | PIK3R2, PTPN11, RAF1 |
| CCR3 Signaling in Eosinophils | 2.12 | PAK1, PIK3R2, RAF1 |
| IL-23 Signaling Pathway | 2.08 | HIF1A, PIK3R2 |
| Th2 Pathway | 2.05 | PIK3R2, RUNX3, TGFBR1 |
| MSP-RON Signaling Pathway | 1.84 | CSF1, PIK3R2 |
| HMGB1 Signaling | 1.83 | FOS, PIK3R2, VCAM1 |
| Th1 and Th2 Activation Pathway | 1.78 | PIK3R2, RUNX3, TGFBR1 |
| CD40 Signaling | 1.76 | FOS, PIK3R2 |
| 3-phosphoinositide Biosynthesis | 1.75 | PIK3R2, PTPN11, RASA1 |
| Mitotic Roles of Polo-Like Kinase | 1.74 | PLK1, WEE1 |
| Regulation of Cellular Mechanics by Calpain Protease | 1.74 | EGFR, PXN |
| Pyridoxal 5'-phosphate Salvage Pathway | 1.64 | PAK1, PLK1 |
| NF-κB Activation by Viruses | 1.56 | PIK3R2, RAF1 |
| Chemokine Signaling | 1.55 | FOS, RAF1 |
| BMP signaling pathway | 1.53 | RAF1, SMAD4 |
| CTLA4 Signaling in Cytotoxic T Lymphocytes | 1.5 | PIK3R2, PTPN11 |
| Superpathway of Inositol Phosphate Compounds | 1.49 | PIK3R2, PTPN11, RASA1 |
| Th17 Activation Pathway | 1.47 | HIF1A, MTOR |
| IL-1 Signaling | 1.46 | ADCY6, FOS |
| Hematopoiesis from Multipotent Stem Cells | 1.44 | CSF1 |
| PPAR Signaling | 1.38 | FOS, RAF1 |
| Salvage Pathways of Pyrimidine Ribonucleotides | 1.31 | PAK1, PLK1 |
| Th1 Pathway | 1.26 | PIK3R2, RUNX3 |
| fMLP Signaling in Neutrophils | 1.25 | PIK3R2, RAF1 |
| GADD45 Signaling | 1.24 | CDKN1A |
| IL-12 Signaling and Production in Macrophages | 1.17 | FOS, PIK3R2 |
| Estrogen Receptor Signaling | 1.17 | ESR1, RAF1 |
| Role of JAK1, JAK2 and TYK2 in Interferon Signaling | 1.14 | RAF1 |
| Role of JAK family kinases in IL-6-type Cytokine Signaling | 1.12 | PTPN11 |
| D-myo-inositol (1, 4, 5, 6)-Tetrakisphosphate Biosynthesis | 1.11 | PTPN11, RASA1 |
| D-myo-inositol (3, 4, 5, 6)-tetrakisphosphate Biosynthesis | 1.11 | PTPN11, RASA1 |
| Apelin Liver Signaling Pathway | 1.11 | IRS1 |
| Epithelial Adherens Junction Signaling | 1.08 | EGFR, TGFBR1 |
| PKCθ Signaling in T Lymphocytes | 1.06 | FOS, PIK3R2 |
| 3-phosphoinositide Degradation | 1.05 | PTPN11, RASA1 |
| D-myo-inositol-5-phosphate Metabolism | 1.04 | PTPN11, RASA1 |
| IL-17A Signaling in Fibroblasts | 0.987 | FOS |
| B Cell Activating Factor Signaling | 0.921 | FOS |
| Production of Nitric Oxide and Reactive Oxygen Species in Macrophages | 0.914 | FOS, PIK3R2 |
| Oncostatin M Signaling | 0.903 | RAF1 |
| MIF Regulation of Innate Immunity | 0.883 | FOS |
| Apelin Pancreas Signaling Pathway | 0.883 | PIK3R2 |
| Role of Oct4 in Mammalian Embryonic Stem Cell Pluripotency | 0.873 | BMI1 |
| iNOS Signaling | 0.857 | FOS |
| Hematopoiesis from Pluripotent Stem Cells | 0.848 | CSF1 |
| Autophagy | 0.735 | MTOR |
| IL-17A Signaling in Airway Cells | 0.73 | PIK3R2 |
| IL-10 Signaling | 0.69 | FOS |
| T Helper Cell Differentiation | 0.686 | TGFBR1 |
| Caveolar-mediated Endocytosis Signaling | 0.68 | EGFR |
| FcγRIIB Signaling in B Lymphocytes | 0.662 | PIK3R2 |
| IL-17 Signaling | 0.656 | PIK3R2 |
| IL-7 Signaling Pathway | 0.656 | PIK3R2 |
| HIPPO signaling | 0.629 | SMAD4 |
| ATM Signaling | 0.583 | CDKN1A |
| CCR5 Signaling in Macrophages | 0.575 | FOS |
| Virus Entry via Endocytic Pathways | 0.527 | PIK3R2 |
| iCOS-iCOSL Signaling in T Helper Cells | 0.517 | PIK3R2 |
| GP6 Signaling Pathway | 0.493 | PIK3R2 |
| Phagosome Formation | 0.491 | PIK3R2 |
| Androgen Signaling | 0.453 | AR |
| Role of Pattern Recognition Receptors in Recognition of Bacteria and Viruses | 0.418 | PIK3R2 |
| Phagosome Maturation | 0.409 | SNAP25 |
| Granulocyte Adhesion and Diapedesis | 0.365 | VCAM1 |
| Dendritic Cell Maturation | 0.36 | PIK3R2 |
| Sertoli Cell-Sertoli Cell Junction Signaling | 0.344 | RAF1 |
| Agranulocyte Adhesion and Diapedesis | 0.344 | VCAM1 |

The enriched pathways were based on the right-tailed Fisher’s exact test (adjusted for False Discover Rate at 5%) that are graphed as negative log p value. These pathways indicate the likelihood of an association of genes to the pathway in MF versus HM fed piglets at different time points.
