# Supplementary material for: Neonatal Diet Impacts Circulatory miRNA Profile in a Porcine Model
Source: Front Immunol. 2020 Jun 23;11:1240. doi: 10.3389/fimmu.2020.01240 (PMC7324749; doi:10.3389/fimmu.2020.01240)
Supplement: Supplementary file 6 [file Table_6.DOCX]

**Table S6. List of genes and enriched pathways of downregulated miRNA in MF compared to HM group at PND 35.**

| **Canonical Pathways** | **-log(p-value)** | **Genes** |
| --- | --- | --- |
| ILK Signaling | 14.3 | CASP3, CCND1, CDH1, DSP, HIF1A, IRS1, ITGB3, MAP2K4, MYC, NFKB1, PDPK1, PIK3R2, PTEN, PTGS2, PXN, RHOB, RHOG, VEGFA, VIM |
| Regulation of the Epithelial-Mesenchymal Transition Pathway | 14.3 | CDH1, FGF16, GRB2, HIF1A, HMGA2, JAK1, JAK2, JAK3, KRAS, MAP2K4, NFKB1, NOTCH1, NRAS, PIK3R2, SMAD3, SMAD4, TGFBR1, WNT1, ZEB2 |
| Senescence Pathway | 12.3 | ANAPC1, BMPR2, CCND1, CDC25A, CDK6, CDKN1A, E2F1, E2F2, E2F3, KRAS, MAP2K4, NFKB1, NRAS, PCGF1, PIK3R2, PTEN, SMAD3, SMAD4, SMAD5, TGFBR1 |
| Estrogen-mediated S-phase Entry | 12.2 | CCND1, CCNE2, CDC25A, CDKN1A, E2F1, E2F2, E2F3, ESR1, MYC |
| IGF-1 Signaling | 11.1 | CCN2, FOXO1, GRB2, IGF1, IRS1, JAK1, JAK2, KRAS, NEDD4, NRAS, PDPK1, PIK3R2, PXN |
| IL-8 Signaling | 10.6 | BAX, BCL2L1, CCND1, CDH1, HMOX1, ITGB3, KRAS, MAP2K4, NFKB1, NRAS, PIK3R2, PTGS2, RHOB, RHOG, VCAM1, VEGFA |
| IL-7 Signaling Pathway | 10.1 | BAX, CCND1, CDC25A, FOXG1, FOXO1, GRB2, JAK1, JAK3, MYC, PDPK1, PIK3R2 |
| STAT3 Pathway | 9.76 | BMPR2, CDC25A, CDKN1A, IGF1, JAK2, KRAS, MAP2K4, MYC, NRAS, NTRK3, PIAS3, TGFBR1, VEGFA |
| PDGF Signaling | 9.39 | ACP1, CRKL, GRB2, JAK1, JAK2, JAK3, KRAS, MAP2K4, MYC, NRAS, PIK3R2 |
| Mouse Embryonic Stem Cell Pluripotency | 8.86 | BMPR2, GRB2, JAK1, JAK2, JAK3, KRAS, MYC, NRAS, PIK3R2, SMAD4, SMAD5 |
| HGF Signaling | 8.39 | CCND1, CDKN1A, CRKL, GRB2, ITGA5, KRAS, MAP2K4, NRAS, PIK3R2, PTGS2, PXN |
| T Cell Exhaustion Signaling Pathway | 8.29 | BMPR2, FOXO1, JAK1, JAK2, JAK3, KRAS, MAP2K4, NRAS, PIK3R2, PRDM1, SMAD3, TGFBR1, VEGFA |
| Role of NANOG in Mammalian Embryonic Stem Cell Pluripotency | 8.19 | BMPR2, GRB2, JAK1, JAK2, JAK3, KRAS, NRAS, PIK3R2, SMAD4, SMAD5, WNT1 |
| Cyclins and Cell Cycle Regulation | 7.5 | CCND1, CCNE2, CDC25A, CDK6, CDKN1A, E2F1, E2F2, E2F3, WEE1 |
| IL-4 Signaling | 7.23 | GRB2, HMGA1, IRS1, JAK1, JAK2, JAK3, KRAS, NRAS, PIK3R2 |
| Oncostatin M Signaling | 7.12 | GRB2, JAK1, JAK2, JAK3, KRAS, MMP13, NRAS |
| Role of JAK1 and JAK3 in γc Cytokine Signaling | 6.87 | GRB2, IRS1, JAK1, JAK2, JAK3, KRAS, NRAS, PIK3R2 |
| GM-CSF Signaling | 6.77 | BCL2L1, CCND1, GRB2, JAK2, KRAS, NRAS, PIK3R2, RUNX1 |
| Th2 Pathway | 6.55 | BMPR2, GRB2, IKZF1, JAK1, JAK2, JAK3, NFKB1, NOTCH1, PIK3R2, TGFBR1 |
| VEGF Signaling | 6.52 | BCL2L1, FOXO1, GRB2, HIF1A, KRAS, NRAS, PIK3R2, PXN, VEGFA |
| Human Embryonic Stem Cell Pluripotency | 6.43 | BMPR2, FOXO1, NTRK3, PDPK1, PIK3R2, SMAD3, SMAD4, SMAD5, TGFBR1, WNT1 |
| mTOR Signaling | 6.42 | EIF3J, EIF4G2, HIF1A, HMOX1, IRS1, KRAS, NRAS, PDPK1, PIK3R2, RHOB, RHOG, VEGFA |
| IL-3 Signaling | 6.41 | CRKL, FOXO1, GRB2, JAK1, JAK2, KRAS, NRAS, PIK3R2 |
| IL-17 Signaling | 6.37 | JAK1, JAK2, KRAS, MAP2K4, NFKB1, NRAS, PIK3R2, PTGS2 |
| Prolactin Signaling | 6.2 | GRB2, IRS1, JAK2, KRAS, MYC, NRAS, PDPK1, PIK3R2 |
| BMP signaling pathway | 6.12 | BMPR2, GRB2, KRAS, MAP2K4, NFKB1, NRAS, SMAD4, SMAD5 |
| B Cell Receptor Signaling | 6.1 | BCL2L1, FOXO1, GRB2, KRAS, MAP2K4, MEF2C, NFKB1, NRAS, PDPK1, PIK3R2, PTEN |
| Regulation of IL-2 Expression in Activated and Anergic T Lymphocytes | 5.94 | GRB2, KRAS, MAP2K4, NFKB1, NRAS, SMAD3, SMAD4, TGFBR1 |
| IL-17A Signaling in Airway Cells | 5.81 | JAK1, JAK2, JAK3, MAP2K4, NFKB1, PIK3R2, PTEN |
| Regulation of Cellular Mechanics by Calpain Protease | 5.81 | CCND1, CDK6, GRB2, ITGA5, KRAS, NRAS, PXN |
| Th1 and Th2 Activation Pathway | 5.61 | BMPR2, GRB2, IKZF1, JAK1, JAK2, JAK3, NFKB1, NOTCH1, PIK3R2, TGFBR1 |
| Germ Cell-Sertoli Cell Junction Signaling | 5.57 | CDH1, KRAS, MAP2K4, NRAS, PDPK1, PIK3R2, PXN, RHOB, RHOG, TGFBR1 |
| iNOS Signaling | 5.48 | HMGA1, JAK1, JAK2, JAK3, NFKB1, TLR4 |
| Paxillin Signaling | 5.37 | GRB2, ITGA5, ITGB3, KRAS, MAP2K4, NRAS, PIK3R2, PXN |
| Erythropoietin Signaling | 5.28 | GRB2, JAK2, KRAS, NFKB1, NRAS, PDPK1, PIK3R2 |
| Production of Nitric Oxide and Reactive Oxygen Species in Macrophages | 5.15 | JAK1, JAK2, JAK3, MAP2K4, NFKB1, PIK3R2, PPP1R7, RHOB, RHOG, TLR4 |
| IL-6 Signaling | 4.9 | GRB2, JAK2, KRAS, MAP2K4, NFKB1, NRAS, PIK3R2, VEGFA |
| HMGB1 Signaling | 4.89 | KRAS, MAP2K4, NFKB1, NRAS, PIK3R2, RHOB, RHOG, TLR4, VCAM1 |
| Cell Cycle Regulation by BTG Family Proteins | 4.82 | CCND1, CCNE2, E2F1, E2F2, E2F3 |
| IL-2 Signaling | 4.78 | GRB2, JAK1, JAK3, KRAS, NRAS, PIK3R2 |
| p70S6K Signaling | 4.66 | F2, GRB2, IRS1, JAK1, KRAS, NRAS, PDPK1, PIK3R2 |
| Thrombopoietin Signaling | 4.63 | GRB2, JAK2, KRAS, MYC, NRAS, PIK3R2 |
| PPAR Signaling | 4.5 | GRB2, KRAS, NFKB1, NRAS, PPARG, PTGS2, RXRA |
| PI3K Signaling in B Lymphocytes | 4.48 | IRS1, KRAS, NFKB1, NRAS, PDPK1, PIK3R2, PTEN, TLR4 |
| IL-23 Signaling Pathway | 4.44 | HIF1A, JAK2, NFKB1, PIK3R2, RUNX1 |
| Clathrin-mediated Endocytosis Signaling | 4.29 | F2, FGF16, GAK, GRB2, IGF1, ITGA5, ITGB3, PIK3R2, VEGFA |
| FcγRIIB Signaling in B Lymphocytes | 4.22 | GRB2, KRAS, MAP2K4, NRAS, PDPK1, PIK3R2 |
| Antiproliferative Role of TOB in T Cell Signaling | 4.17 | CCNE2, SMAD3, SMAD4, TGFBR1 |
| PEDF Signaling | 4.13 | BCL2L1, KRAS, NFKB1, NRAS, PIK3R2, PPARG |
| NF-κB Activation by Viruses | 4.1 | ITGA5, ITGB3, KRAS, NFKB1, NRAS, PIK3R2 |
| Th1 Pathway | 4.05 | GRB2, JAK1, JAK2, JAK3, NFKB1, NOTCH1, PIK3R2 |
| HIPPO signaling | 4.01 | CSNK1D, NF2, PPP1R7, SMAD3, SMAD4, SMAD5 |
| Role of CHK Proteins in Cell Cycle Checkpoint Control | 3.9 | CDC25A, CDKN1A, E2F1, E2F2, E2F3 |
| Actin Cytoskeleton Signaling | 3.88 | CRKL, F2, FGF16, GRB2, ITGA5, KRAS, NRAS, PIK3R2, PXN |
| 3-phosphoinositide Biosynthesis | 3.83 | ACP1, CDC25A, CDIPT, DUSP12, DUSP23, PIK3R2, PPP1R7, PTEN |
| Th17 Activation Pathway | 3.82 | HIF1A, JAK1, JAK2, JAK3, NFKB1, RUNX1 |
| UVA-Induced MAPK Signaling | 3.68 | BCL2L1, CASP3, KRAS, MAP2K4, NRAS, PIK3R2 |
| CD40 Signaling | 3.62 | JAK3, MAP2K4, NFKB1, PIK3R2, PTGS2 |
| Mitotic Roles of Polo-Like Kinase | 3.59 | ANAPC1, CDC25A, PKMYT1, SMC1A, WEE1 |
| Sumoylation Pathway | 3.45 | CDH1, MAP2K4, NFKB1, RHOB, RHOG, SMAD4 |
| Epithelial Adherens Junction Signaling | 3.43 | BMPR2, CDH1, KRAS, NOTCH1, NRAS, PTEN, TGFBR1 |
| T Cell Receptor Signaling | 3.43 | GRB2, KRAS, MAP2K4, NFKB1, NRAS, PIK3R2 |
| Leukocyte Extravasation Signaling | 3.43 | CRKL, ITGA5, ITGB3, MAP2K4, MMP13, PIK3R2, PXN, VCAM1 |
| Growth Hormone Signaling | 3.33 | IGF1, IRS1, JAK2, PDPK1, PIK3R2 |
| TREM1 Signaling | 3.31 | GRB2, ITGA5, JAK2, NFKB1, TLR4 |
| Macropinocytosis Signaling | 3.31 | ITGA5, ITGB3, KRAS, NRAS, PIK3R2 |
| GADD45 Signaling | 3.27 | CCND1, CCNE2, CDKN1A |
| MIF Regulation of Innate Immunity | 3.23 | MAP2K4, NFKB1, PTGS2, TLR4 |
| FLT3 Signaling in Hematopoietic Progenitor Cells | 3.21 | GRB2, KRAS, NRAS, PDPK1, PIK3R2 |
| Fc Epsilon RI Signaling | 3.17 | GRB2, KRAS, MAP2K4, NRAS, PDPK1, PIK3R2 |
| Superpathway of Inositol Phosphate Compounds | 3.17 | ACP1, CDC25A, CDIPT, DUSP12, DUSP23, PIK3R2, PPP1R7, PTEN |
| CXCR4 Signaling | 3.1 | KRAS, MAP2K4, NRAS, PIK3R2, PXN, RHOB, RHOG |
| VEGF Family Ligand-Receptor Interactions | 2.97 | GRB2, KRAS, NRAS, PIK3R2, VEGFA |
| Role of JAK1, JAK2 and TYK2 in Interferon Signaling | 2.96 | JAK1, JAK2, NFKB1 |
| IL-12 Signaling and Production in Macrophages | 2.94 | MAP2K4, NFKB1, PIK3R2, PPARG, RXRA, TLR4 |
| Estrogen Receptor Signaling | 2.93 | ESR1, GRB2, KRAS, NRAS, POLR2C, TAF9B |
| Role of JAK family kinases in IL-6-type Cytokine Signaling | 2.91 | JAK1, JAK2, MAP2K4 |
| Apelin Liver Signaling Pathway | 2.86 | COL1A2, IRS1, MAP2K4 |
| ATM Signaling | 2.83 | CDC25A, CDKN1A, FANCD2, MAP2K4, SMC1A |
| MSP-RON Signaling Pathway | 2.79 | JAK2, KLK10, PIK3R2, TLR4 |
| EGF Signaling | 2.79 | GRB2, JAK1, MAP2K4, PIK3R2 |
| D-myo-inositol (1, 4, 5, 6)-Tetrakisphosphate Biosynthesis | 2.77 | ACP1, CDC25A, DUSP12, DUSP23, PPP1R7, PTEN |
| D-myo-inositol (3, 4, 5, 6)-tetrakisphosphate Biosynthesis | 2.77 | ACP1, CDC25A, DUSP12, DUSP23, PPP1R7, PTEN |
| PKCθ Signaling in T Lymphocytes | 2.6 | GRB2, KRAS, MAP2K4, NFKB1, NRAS, PIK3R2 |
| 3-phosphoinositide Degradation | 2.57 | ACP1, CDC25A, DUSP12, DUSP23, PPP1R7, PTEN |
| D-myo-inositol-5-phosphate Metabolism | 2.56 | ACP1, CDC25A, DUSP12, DUSP23, PPP1R7, PTEN |
| Virus Entry via Endocytic Pathways | 2.54 | ITGA5, ITGB3, KRAS, NRAS, PIK3R2 |
| Role of JAK2 in Hormone-like Cytokine Signaling | 2.52 | IRS1, JAK1, JAK2 |
| Antioxidant Action of Vitamin C | 2.5 | HMOX1, JAK2, MAP2K4, NFKB1, NXN |
| iCOS-iCOSL Signaling in T Helper Cells | 2.49 | GRB2, NFKB1, PDPK1, PIK3R2, PTEN |
| IL-10 Signaling | 2.45 | HMOX1, JAK1, MAP2K4, NFKB1 |
| MIF-mediated Glucocorticoid Regulation | 2.45 | NFKB1, PTGS2, TLR4 |
| Interferon Signaling | 2.45 | BAX, JAK1, JAK2 |
| GP6 Signaling Pathway | 2.36 | COL1A2, ITGB3, PDPK1, PIK3R2, RHOG |
| CD28 Signaling in T Helper Cells | 2.35 | GRB2, MAP2K4, NFKB1, PDPK1, PIK3R2 |
| Phagosome Formation | 2.35 | ITGA5, PIK3R2, RHOB, RHOG, TLR4 |
| Dendritic Cell Maturation | 2.29 | COL1A2, JAK2, MAP2K4, NFKB1, PIK3R2, TLR4 |
| Role of PKR in Interferon Induction and Antiviral Response | 2.29 | CASP3, FADD, NFKB1 |
| Antiproliferative Role of Somatostatin Receptor 2 | 2.29 | CDKN1A, KRAS, NRAS, PIK3R2 |
| Sertoli Cell-Sertoli Cell Junction Signaling | 2.19 | CDH1, ITGA5, KRAS, MAP2K4, NRAS, PTEN |
| Apelin Pancreas Signaling Pathway | 2.17 | MAP2K4, NFKB1, PIK3R2 |
| FGF Signaling | 2.16 | CRKL, FGF16, GRB2, PIK3R2 |
| Role of Oct4 in Mammalian Embryonic Stem Cell Pluripotency | 2.15 | IGF2BP1, PHB, REST |
| Melanocyte Development and Pigmentation Signaling | 2.03 | GRB2, KRAS, NRAS, PIK3R2 |
| UVC-Induced MAPK Signaling | 2.02 | KRAS, MAP2K4, NRAS |
| Fcγ Receptor-mediated Phagocytosis in Macrophages and Monocytes | 1.97 | HMOX1, PIK3R2, PTEN, PXN |
| Activation of IRF by Cytosolic Pattern Recognition Receptors | 1.77 | FADD, MAP2K4, NFKB1 |
| IL-22 Signaling | 1.75 | JAK1, MAP2K4 |
| IL-17A Signaling in Gastric Cells | 1.72 | MAP2K4, NFKB1 |
| fMLP Signaling in Neutrophils | 1.67 | KRAS, NFKB1, NRAS, PIK3R2 |
| Natural Killer Cell Signaling | 1.66 | GRB2, KRAS, NRAS, PIK3R2 |
| Pyridoxal 5'-phosphate Salvage Pathway | 1.58 | CDK6, CSNK1D, MAP2K4 |
| 4-1BB Signaling in T Lymphocytes | 1.52 | MAP2K4, NFKB1 |
| Androgen Signaling | 1.5 | CCND1, NFKB1, POLR2C, SMAD3 |
| Putrescine Biosynthesis III | 1.47 | ODC1 |
| White Adipose Tissue Browning Pathway | 1.46 | PPARG, RXRA, THRB, VEGFA |
| Gap Junction Signaling | 1.45 | CSNK1D, GRB2, KRAS, NRAS, PIK3R2 |
| CTLA4 Signaling in Cytotoxic T Lymphocytes | 1.39 | GRB2, JAK2, PIK3R2 |
| OX40 Signaling Pathway | 1.38 | BCL2L1, MAP2K4, NFKB1 |
| Role of Pattern Recognition Receptors in Recognition of Bacteria and Viruses | 1.36 | MAP2K4, NFKB1, PIK3R2, TLR4 |
| B Cell Activating Factor Signaling | 1.32 | MAP2K4, NFKB1 |
| Mechanisms of Viral Exit from Host Cells | 1.32 | CHMP2A, NEDD4 |
| Relaxin Signaling | 1.31 | ENPP6, NFKB1, PIK3R2, VEGFA |
| Sperm Motility | 1.27 | JAK1, JAK2, JAK3, MAP2K4, NTRK3 |
| Role of RIG1-like Receptors in Antiviral Innate Immunity | 1.26 | FADD, NFKB1 |
| Spermidine Biosynthesis I | 1.23 | SRM |
| NER Pathway | 1.19 | POLD2, POLR2C, PRIM1 |
| Assembly of RNA Polymerase II Complex | 1.17 | POLR2C, TAF9B |
| Granulocyte Adhesion and Diapedesis | 1.15 | ITGA5, ITGB3, MMP13, VCAM1 |
| UVB-Induced MAPK Signaling | 1.14 | MAP2K4, PIK3R2 |
| Thioredoxin Pathway | 1.13 | NXN |
| Salvage Pathways of Pyrimidine Ribonucleotides | 1.13 | CDK6, CSNK1D, MAP2K4 |
| Cell Cycle Control of Chromosomal Replication | 1.08 | CDK6, PRIM1 |
| Heme Degradation | 1.04 | HMOX1 |
| Melatonin Degradation II | 1.01 | SMOX |
| Glycogen Biosynthesis II (from UDP-D-Glucose) | 1.01 | GYS1 |
| CCR3 Signaling in Eosinophils | 1 | KRAS, NRAS, PIK3R2 |
| Spermine and Spermidine Degradation I | 0.947 | SMOX |
| dTMP De Novo Biosynthesis | 0.947 | TYMS |
| Remodeling of Epithelial Adherens Junctions | 0.939 | CDH1, MAPRE1 |
| Granzyme B Signaling | 0.893 | CASP3 |
| Prostanoid Biosynthesis | 0.893 | PTGS2 |
| Caveolar-mediated Endocytosis Signaling | 0.87 | ITGA5, ITGB3 |
| Corticotropin Releasing Hormone Signaling | 0.83 | MEF2C, PTGS2, VEGFA |
| Oleate Biosynthesis II (Animals) | 0.821 | FADS2 |
| DNA damage-induced 14-3-3σ Signaling | 0.821 | CCNE2 |
| Phagosome Maturation | 0.812 | ATP6V0A1, ATP6V1F, VPS39 |
| NAD Phosphorylation and Dephosphorylation | 0.801 | ACP1 |
| Chemokine Signaling | 0.79 | KRAS, NRAS |
| Endoplasmic Reticulum Stress Pathway | 0.783 | CASP3 |
| Superpathway of Melatonin Degradation | 0.775 | CYP1B1, SMOX |
| Chondroitin Sulfate Degradation (Metazoa) | 0.747 | CEMIP2 |
| Dermatan Sulfate Degradation (Metazoa) | 0.747 | CEMIP2 |
| Fatty Acid α-oxidation | 0.747 | PTGS2 |
| Vitamin-C Transport | 0.747 | NXN |
| Crosstalk between Dendritic Cells and Natural Killer Cells | 0.745 | NFKB1, TLR4 |
| γ-linolenate Biosynthesis II (Animals) | 0.73 | FADS2 |
| Choline Biosynthesis III | 0.73 | HMOX1 |
| D-myo-inositol (1, 3, 4)-trisphosphate Biosynthesis | 0.73 | PTEN |
| IL-1 Signaling | 0.717 | MAP2K4, NFKB1 |
| Bupropion Degradation | 0.684 | CYP1B1 |
| Agranulocyte Adhesion and Diapedesis | 0.64 | ITGA5, MMP13, VCAM1 |
| The Visual Cycle | 0.62 | RDH10 |
| Superpathway of D-myo-inositol (1, 4, 5)-trisphosphate Metabolism | 0.62 | PTEN |
| Putrescine Degradation III | 0.607 | SMOX |
| Phenylalanine Degradation IV (Mammalian, via Side Chain) | 0.597 | SMOX |
| Coagulation System | 0.585 | F2 |
| IL-17A Signaling in Fibroblasts | 0.585 | NFKB1 |
| NAD Salvage Pathway II | 0.585 | ACP1 |
| D-myo-inositol (1, 4, 5)-Trisphosphate Biosynthesis | 0.585 | CDIPT |
| Tryptophan Degradation X (Mammalian, via Tryptamine) | 0.585 | SMOX |
| Nucleotide Excision Repair Pathway | 0.585 | POLR2C |
| Retinoate Biosynthesis I | 0.565 | RDH10 |
| Acetone Degradation I (to Methylglyoxal) | 0.565 | CYP1B1 |
| Pyrimidine Deoxyribonucleotides De Novo Biosynthesis I | 0.536 | TYMS |
| Inhibition of Matrix Metalloproteases | 0.536 | MMP13 |
| Dopamine Degradation | 0.502 | SMOX |
| tRNA Splicing | 0.457 | ENPP6 |
| Retinol Biosynthesis | 0.45 | RDH10 |
| Estrogen Biosynthesis | 0.442 | CYP1B1 |
| Noradrenaline and Adrenaline Degradation | 0.442 | SMOX |
| Triacylglycerol Degradation | 0.437 | AARSD1 |
| Transcriptional Regulatory Network in Embryonic Stem Cells | 0.431 | REST |
| Nicotine Degradation III | 0.389 | CYP1B1 |
| Cdc42 Signaling | 0.372 | ITGA5, MAP2K4 |
| Autophagy | 0.369 | VPS39 |
| Melatonin Degradation I | 0.34 | CYP1B1 |
| Phospholipases | 0.332 | HMOX1 |
| T Helper Cell Differentiation | 0.327 | TGFBR1 |
| Nicotine Degradation II | 0.323 | CYP1B1 |
| Heparan Sulfate Biosynthesis (Late Stages) | 0.303 | AARSD1 |
| tRNA Charging | 0.296 | MARS2 |
| Serotonin Degradation | 0.288 | SMOX |
| Heparan Sulfate Biosynthesis | 0.265 | AARSD1 |
| Communication between Innate and Adaptive Immune Cells | 0.247 | TLR4 |
| CCR5 Signaling in Macrophages | 0.242 | MAP2K4 |

The enriched pathways were based on the right-tailed Fisher’s exact test (adjusted for False Discover Rate at 5%) that are graphed as negative log p value. These pathways indicate the likelihood of an association of genes to the pathway in MF versus HM fed piglets at different time points.
