# Supplementary material for: Neonatal Diet Impacts Circulatory miRNA Profile in a Porcine Model
Source: Front Immunol. 2020 Jun 23;11:1240. doi: 10.3389/fimmu.2020.01240 (PMC7324749; doi:10.3389/fimmu.2020.01240)
Supplement: Supplementary file 7 [file Table_7.DOCX]

**Table S7. List of genes and enriched pathways of downregulated miRNA in MF compared to HM group at PND 51.**

| **Canonical Pathways** | **-log(p-value)** | **Genes** |
| --- | --- | --- |
| Estrogen-mediated S-phase Entry | 16 | CCNA2, CDK1, CDK4, CDKN1A, E2F1, E2F2, E2F3, ESR1, MYC |
| Senescence Pathway | 13.7 | ACVR1B, CDK1, CDK4, CDKN1A, CDKN2A, E2F1, E2F2, E2F3, MAP2K4, MAPK14, MTOR, PTEN, SMAD3, SMAD4, SMAD5 |
| Cyclins and Cell Cycle Regulation | 11.2 | CCNA2, CDK1, CDK4, CDKN1A, CDKN2A, E2F1, E2F2, E2F3, WEE1 |
| STAT3 Pathway | 9.14 | CDKN1A, FGFR3, IGF1, IGF1R, IL6R, MAP2K4, MAPK14, MYC, PIAS3 |
| Role of CHK Proteins in Cell Cycle Checkpoint Control | 9.11 | BRCA1, CDK1, CDKN1A, E2F1, E2F2, E2F3, PLK1 |
| IL-7 Signaling Pathway | 8.05 | BAX, FOXO1, GRB2, MAPK14, MET, MYC, PDPK1 |
| Regulation of the Epithelial-Mesenchymal Transition Pathway | 7.82 | FGF16, FGFR3, GRB2, HIF1A, MAP2K4, MET, NOTCH1, SMAD3, SMAD4 |
| IGF-1 Signaling | 7.2 | CCN2, FOXO1, GRB2, IGF1, IGF1R, PDPK1, PXN |
| B Cell Receptor Signaling | 6.64 | FOXO1, GRB2, MAP2K4, MAPK14, MEF2C, MTOR, PDPK1, PTEN |
| GADD45 Signaling | 6.38 | BRCA1, CDK1, CDK4, CDKN1A |
| HGF Signaling | 5.66 | CDKN1A, CDKN2A, GRB2, MAP2K4, MET, PXN |
| Regulation of Cellular Mechanics by Calpain Protease | 5.57 | CCNA2, CDK1, CDK4, GRB2, PXN |
| ILK Signaling | 5.45 | HIF1A, MAP2K4, MTOR, MYC, PDPK1, PTEN, PXN |
| Cell Cycle Regulation by BTG Family Proteins | 5.17 | CDK4, E2F1, E2F2, E2F3 |
| Human Embryonic Stem Cell Pluripotency | 5.14 | FGFR3, FOXO1, PDPK1, SMAD3, SMAD4, SMAD5 |
| BMP signaling pathway | 5 | GRB2, MAP2K4, MAPK14, SMAD4, SMAD5 |
| FGF Signaling | 4.95 | FGF16, FGFR3, GRB2, MAPK14, MET |
| ATM Signaling | 4.75 | BRCA1, CDK1, CDKN1A, MAP2K4, MAPK14 |
| Mouse Embryonic Stem Cell Pluripotency | 4.62 | GRB2, MAPK14, MYC, SMAD4, SMAD5 |
| T Cell Exhaustion Signaling Pathway | 4.53 | ACVR1B, FOXO1, IL6R, MAP2K4, MTOR, SMAD3 |
| EGF Signaling | 4.35 | GRB2, MAP2K4, MAPK14, MTOR |
| Mitotic Roles of Polo-Like Kinase | 4.16 | CDK1, PKMYT1, PLK1, WEE1 |
| Role of JAK family kinases in IL-6-type Cytokine Signaling | 4.12 | IL6R, MAP2K4, MAPK14 |
| Antiproliferative Role of TOB in T Cell Signaling | 4.06 | CCNA2, SMAD3, SMAD4 |
| Pyridoxal 5'-phosphate Salvage Pathway | 3.94 | CDK1, CDK4, MAP2K4, PLK1 |
| White Adipose Tissue Browning Pathway | 3.94 | FGFR3, MAPK14, PPARG, RXRA, THRB |
| FLT3 Signaling in Hematopoietic Progenitor Cells | 3.84 | GRB2, MAPK14, MTOR, PDPK1 |
| Regulation of IL-2 Expression in Activated and Anergic T Lymphocytes | 3.62 | GRB2, MAP2K4, SMAD3, SMAD4 |
| Th17 Activation Pathway | 3.58 | HIF1A, IL6R, MTOR, RUNX1 |
| Oncostatin M Signaling | 3.4 | GRB2, MMP13, PLAU |
| VEGF Signaling | 3.38 | FOXO1, GRB2, HIF1A, PXN |
| Paxillin Signaling | 3.34 | GRB2, MAP2K4, MAPK14, PXN |
| Salvage Pathways of Pyrimidine Ribonucleotides | 3.25 | CDK1, CDK4, MAP2K4, PLK1 |
| Leukocyte Extravasation Signaling | 3.24 | CXCL12, MAP2K4, MAPK14, MMP13, PXN |
| UVB-Induced MAPK Signaling | 3.16 | MAP2K4, MAPK14, MTOR |
| Fc Epsilon RI Signaling | 3.12 | GRB2, MAP2K4, MAPK14, PDPK1 |
| IL-6 Signaling | 3.1 | GRB2, IL6R, MAP2K4, MAPK14 |
| IL-12 Signaling and Production in Macrophages | 2.96 | MAP2K4, MAPK14, PPARG, RXRA |
| IL-17A Signaling in Airway Cells | 2.86 | MAP2K4, MAPK14, PTEN |
| Epithelial Adherens Junction Signaling | 2.76 | ACVR1B, MET, NOTCH1, PTEN |
| DNA damage-induced 14-3-3σ Signaling | 2.75 | BRCA1, CDK1 |
| Growth Hormone Signaling | 2.7 | IGF1, IGF1R, PDPK1 |
| FcγRIIB Signaling in B Lymphocytes | 2.64 | GRB2, MAP2K4, PDPK1 |
| Th1 and Th2 Activation Pathway | 2.59 | ACVR1B, GRB2, IL6R, NOTCH1 |
| Germ Cell-Sertoli Cell Junction Signaling | 2.57 | MAP2K4, MAPK14, PDPK1, PXN |
| Prolactin Signaling | 2.56 | GRB2, MYC, PDPK1 |
| IL-22 Signaling | 2.54 | MAP2K4, MAPK14 |
| HIPPO signaling | 2.53 | SMAD3, SMAD4, SMAD5 |
| IL-17A Signaling in Gastric Cells | 2.51 | MAP2K4, MAPK14 |
| PDGF Signaling | 2.45 | GRB2, MAP2K4, MYC |
| Clathrin-mediated Endocytosis Signaling | 2.38 | FGF16, GRB2, IGF1, MET |
| UVA-Induced MAPK Signaling | 2.36 | MAP2K4, MAPK14, MTOR |
| PPAR Signaling | 2.3 | GRB2, PPARG, RXRA |
| 4-1BB Signaling in T Lymphocytes | 2.3 | MAP2K4, MAPK14 |
| mTOR Signaling | 2.24 | HIF1A, MTOR, PDPK1, RPTOR |
| iCOS-iCOSL Signaling in T Helper Cells | 2.17 | GRB2, PDPK1, PTEN |
| Role of NANOG in Mammalian Embryonic Stem Cell Pluripotency | 2.14 | GRB2, SMAD4, SMAD5 |
| Th1 Pathway | 2.11 | GRB2, IL6R, NOTCH1 |
| Sperm Motility | 2.09 | FGFR3, IGF1R, MAP2K4, MET |
| Role of PKR in Interferon Induction and Antiviral Response | 2.09 | FADD, MAPK14 |
| B Cell Activating Factor Signaling | 2.09 | MAP2K4, MAPK14 |
| CD28 Signaling in T Helper Cells | 2.08 | GRB2, MAP2K4, PDPK1 |
| IL-23 Signaling Pathway | 2.03 | HIF1A, RUNX1 |
| Role of Oct4 in Mammalian Embryonic Stem Cell Pluripotency | 1.99 | BRCA1, PHB |
| p70S6K Signaling | 1.99 | GRB2, MTOR, PDPK1 |
| Th2 Pathway | 1.98 | ACVR1B, GRB2, NOTCH1 |
| UVC-Induced MAPK Signaling | 1.9 | MAP2K4, MAPK14 |
| Putrescine Biosynthesis III | 1.88 | ODC1 |
| Cell Cycle Control of Chromosomal Replication | 1.83 | CDK1, CDK4 |
| Activation of IRF by Cytosolic Pattern Recognition Receptors | 1.73 | FADD, MAP2K4 |
| CD40 Signaling | 1.71 | MAP2K4, MAPK14 |
| CXCR4 Signaling | 1.69 | CXCL12, MAP2K4, PXN |
| Thrombopoietin Signaling | 1.68 | GRB2, MYC |
| Spermidine Biosynthesis I | 1.64 | SRM |
| GM-CSF Signaling | 1.63 | GRB2, RUNX1 |
| IL-10 Signaling | 1.61 | MAP2K4, MAPK14 |
| Sertoli Cell-Sertoli Cell Junction Signaling | 1.58 | MAP2K4, MAPK14, PTEN |
| Erythropoietin Signaling | 1.55 | GRB2, PDPK1 |
| IL-3 Signaling | 1.55 | FOXO1, GRB2 |
| IL-17 Signaling | 1.54 | MAP2K4, MAPK14 |
| IL-8 Signaling | 1.52 | BAX, MAP2K4, MTOR |
| PEDF Signaling | 1.52 | MAPK14, PPARG |
| Chemokine Signaling | 1.5 | CXCL12, MAPK14 |
| IL-4 Signaling | 1.47 | GRB2, MTOR |
| Actin Cytoskeleton Signaling | 1.42 | FGF16, GRB2, PXN |
| IL-1 Signaling | 1.41 | MAP2K4, MAPK14 |
| BER pathway | 1.37 | FEN1 |
| CCR5 Signaling in Macrophages | 1.37 | MAP2K4, MAPK14 |
| Fcγ Receptor-mediated Phagocytosis in Macrophages and Monocytes | 1.36 | PTEN, PXN |
| DNA Double-Strand Break Repair by Homologous Recombination | 1.34 | BRCA1 |
| dTMP De Novo Biosynthesis | 1.34 | DHFR |
| Aspartate Degradation II | 1.34 | MDH2 |
| Sumoylation Pathway | 1.29 | MAP2K4, SMAD4 |
| T Cell Receptor Signaling | 1.29 | GRB2, MAP2K4 |
| Granzyme B Signaling | 1.29 | LMNB1 |
| Antioxidant Action of Vitamin C | 1.25 | MAP2K4, MAPK14 |
| Mismatch Repair in Eukaryotes | 1.24 | FEN1 |
| Estrogen Receptor Signaling | 1.12 | ESR1, GRB2 |
| D-myo-inositol (1, 3, 4)-trisphosphate Biosynthesis | 1.12 | PTEN |
| PI3K Signaling in B Lymphocytes | 1.09 | PDPK1, PTEN |
| Apelin Liver Signaling Pathway | 1.08 | MAP2K4 |
| Bupropion Degradation | 1.07 | CYP1B1 |
| Corticotropin Releasing Hormone Signaling | 1.03 | MAPK14, MEF2C |
| PKCθ Signaling in T Lymphocytes | 1.01 | GRB2, MAP2K4 |
| Superpathway of D-myo-inositol (1, 4, 5)-trisphosphate Metabolism | 0.996 | PTEN |
| HMGB1 Signaling | 0.983 | MAP2K4, MAPK14 |
| Cdc42 Signaling | 0.967 | MAP2K4, MAPK14 |
| Coagulation System | 0.959 | PLAU |
| IL-17A Signaling in Fibroblasts | 0.959 | MAPK14 |
| Interferon Signaling | 0.947 | BAX |
| Acetone Degradation I (to Methylglyoxal) | 0.936 | CYP1B1 |
| Granulocyte Adhesion and Diapedesis | 0.917 | CXCL12, MMP13 |
| Dendritic Cell Maturation | 0.907 | MAP2K4, MAPK14 |
| Inhibition of Matrix Metalloproteases | 0.907 | MMP13 |
| Mechanisms of Viral Exit from Host Cells | 0.896 | LMNB1 |
| TCA Cycle II (Eukaryotic) | 0.886 | MDH2 |
| Agranulocyte Adhesion and Diapedesis | 0.873 | CXCL12, MMP13 |
| Role of RIG1-like Receptors in Antiviral Innate Immunity | 0.866 | FADD |
| Production of Nitric Oxide and Reactive Oxygen Species in Macrophages | 0.866 | MAP2K4, MAPK14 |
| MIF Regulation of Innate Immunity | 0.857 | MAP2K4 |
| Apelin Pancreas Signaling Pathway | 0.857 | MAP2K4 |
| Gluconeogenesis I | 0.839 | MDH2 |
| iNOS Signaling | 0.833 | MAPK14 |
| Estrogen Biosynthesis | 0.799 | CYP1B1 |
| Nicotine Degradation III | 0.735 | CYP1B1 |
| IL-2 Signaling | 0.724 | GRB2 |
| Autophagy | 0.712 | MTOR |
| Remodeling of Epithelial Adherens Junctions | 0.695 | MET |
| Role of JAK1 and JAK3 in γc Cytokine Signaling | 0.688 | GRB2 |
| Melatonin Degradation I | 0.678 | CYP1B1 |
| T Helper Cell Differentiation | 0.662 | IL6R |
| Nicotine Degradation II | 0.656 | CYP1B1 |
| TREM1 Signaling | 0.652 | GRB2 |
| Macropinocytosis Signaling | 0.652 | MET |
| Antiproliferative Role of Somatostatin Receptor 2 | 0.627 | CDKN1A |
| Superpathway of Melatonin Degradation | 0.604 | CYP1B1 |
| CTLA4 Signaling in Cytotoxic T Lymphocytes | 0.592 | GRB2 |
| OX40 Signaling Pathway | 0.587 | MAP2K4 |
| VEGF Family Ligand-Receptor Interactions | 0.587 | GRB2 |
| Melanocyte Development and Pigmentation Signaling | 0.564 | GRB2 |
| Natural Killer Cell Signaling | 0.471 | GRB2 |
| GP6 Signaling Pathway | 0.471 | PDPK1 |
| CCR3 Signaling in Eosinophils | 0.457 | MAPK14 |
| Androgen Signaling | 0.431 | SMAD3 |
| D-myo-inositol (1, 4, 5, 6)-Tetrakisphosphate Biosynthesis | 0.412 | PTEN |
| D-myo-inositol (3, 4, 5, 6)-tetrakisphosphate Biosynthesis | 0.412 | PTEN |
| Role of Pattern Recognition Receptors in Recognition of Bacteria and Viruses | 0.397 | MAP2K4 |
| 3-phosphoinositide Degradation | 0.382 | PTEN |
| D-myo-inositol-5-phosphate Metabolism | 0.38 | PTEN |
| 3-phosphoinositide Biosynthesis | 0.351 | PTEN |
| Gap Junction Signaling | 0.297 | GRB2 |
| Superpathway of Inositol Phosphate Compounds | 0.281 | PTEN |

The enriched pathways were based on the right-tailed Fisher’s exact test (adjusted for False Discover Rate at 5%) that are graphed as negative log p value. These pathways indicate the likelihood of an association of genes to the pathway in MF versus HM fed piglets at different time points.
