# Supplementary material for: Neonatal Diet Impacts Circulatory miRNA Profile in a Porcine Model
Source: Front Immunol. 2020 Jun 23;11:1240. doi: 10.3389/fimmu.2020.01240 (PMC7324749; doi:10.3389/fimmu.2020.01240)
Supplement: Supplementary file 8 [file Table_8.DOCX]

**Table S8. List of genes and enriched pathways of upregulated miRNA in MF compared to HM group at PND 51.**

| **Canonical Pathways** | **-log(p-value)** | **Genes** |
| --- | --- | --- |
| Regulation of the Epithelial-Mesenchymal Transition Pathway | 11.1 | ETS1, FGF16, FGFR3, HIF1A, HMGA2, MET, MMP9, RAP1A, RAP1B, ZEB1, ZEB2 |
| B Cell Receptor Signaling | 9.77 | BCL6, CREB1, ETS1, INPPL1, MTOR, PTEN, RAP1A, RAP1B, SHC1, VAV2 |
| ILK Signaling | 9.73 | CREB1, FN1, HIF1A, ITGB4, MMP9, MTOR, PTEN, PTGS2, VEGFA, VIM |
| Senescence Pathway | 6.97 | CDC25B, E2F1, ETS1, MTOR, PTEN, RAP1A, RAP1B, SIRT1, SMAD1 |
| HGF Signaling | 6.03 | ETS1, MET, PRKCE, PTGS2, RAP1A, RAP1B |
| Natural Killer Cell Signaling | 5.82 | INPPL1, PRKCE, RAP1A, RAP1B, SHC1, VAV2 |
| GM-CSF Signaling | 5.73 | ETS1, RAP1A, RAP1B, RUNX1, SHC1 |
| Leukocyte Extravasation Signaling | 5.72 | CD44, MMP3, MMP9, PRKCE, RAP1A, RAP1B, VAV2 |
| IL-8 Signaling | 5.71 | MMP9, MTOR, PRKCE, PTGS2, RAP1A, RAP1B, VEGFA |
| Macropinocytosis Signaling | 5.58 | ITGB4, MET, PRKCE, RAP1A, RAP1B |
| mTOR Signaling | 5.54 | HIF1A, MTOR, PRKCE, RAP1A, RAP1B, RPTOR, VEGFA |
| FLT3 Signaling in Hematopoietic Progenitor Cells | 5.47 | CREB1, MTOR, RAP1A, RAP1B, SHC1 |
| Actin Cytoskeleton Signaling | 5.46 | ARPC1B, FGF16, FN1, RAP1A, RAP1B, SHC1, VAV2 |
| BMP signaling pathway | 5.31 | BMP1, CREB1, RAP1A, RAP1B, SMAD1 |
| IL-4 Signaling | 5.29 | INPPL1, MTOR, RAP1A, RAP1B, SHC1 |
| Epithelial Adherens Junction Signaling | 5.27 | ARPC1B, MET, PTEN, RAP1A, RAP1B, VAV2 |
| Corticotropin Releasing Hormone Signaling | 5.26 | CREB1, PRKCE, PTGS2, RAP1A, RAP1B, VEGFA |
| VEGF Family Ligand-Receptor Interactions | 5.22 | PRKCE, RAP1A, RAP1B, SHC1, VEGFA |
| Oncostatin M Signaling | 5.15 | MMP3, RAP1A, RAP1B, SHC1 |
| PPAR Signaling | 4.91 | MED1, PTGS2, RAP1A, RAP1B, SHC1 |
| VEGF Signaling | 4.89 | HIF1A, RAP1A, RAP1B, SHC1, VEGFA |
| Role of NANOG in Mammalian Embryonic Stem Cell Pluripotency | 4.63 | BMP1, RAP1A, RAP1B, SHC1, SMAD1 |
| Fc Epsilon RI Signaling | 4.56 | INPPL1, PRKCE, RAP1A, RAP1B, VAV2 |
| Thrombopoietin Signaling | 4.38 | PRKCE, RAP1A, RAP1B, SHC1 |
| p70S6K Signaling | 4.36 | MTOR, PRKCE, RAP1A, RAP1B, SHC1 |
| STAT3 Pathway | 4.35 | FGFR3, IGF1R, RAP1A, RAP1B, VEGFA |
| PI3K Signaling in B Lymphocytes | 4.24 | CREB1, PTEN, RAP1A, RAP1B, VAV2 |
| Sperm Motility | 4.2 | DYRK1A, ERBB3, FGFR3, IGF1R, MET, PRKCE |
| Erythropoietin Signaling | 4.1 | PRKCE, RAP1A, RAP1B, SHC1 |
| IL-3 Signaling | 4.1 | PRKCE, RAP1A, RAP1B, SHC1 |
| IL-17 Signaling | 4.08 | MMP3, PTGS2, RAP1A, RAP1B |
| Relaxin Signaling | 4.01 | CREB1, MMP9, RAP1A, RAP1B, VEGFA |
| Prolactin Signaling | 4 | PRKCE, RAP1A, RAP1B, SHC1 |
| 3-phosphoinositide Degradation | 4 | ATP1A1, CDC25B, INPPL1, PTEN, SET |
| FGF Signaling | 3.92 | CREB1, FGF16, FGFR3, MET |
| PDGF Signaling | 3.84 | INPPL1, RAP1A, RAP1B, SHC1 |
| T Cell Exhaustion Signaling Pathway | 3.79 | BCL6, MTOR, RAP1A, RAP1B, VEGFA |
| Melanocyte Development and Pigmentation Signaling | 3.77 | CREB1, RAP1A, RAP1B, SHC1 |
| Fcγ Receptor-mediated Phagocytosis in Macrophages and Monocytes | 3.71 | ARPC1B, PRKCE, PTEN, VAV2 |
| IGF-1 Signaling | 3.61 | IGF1R, RAP1A, RAP1B, SHC1 |
| Clathrin-mediated Endocytosis Signaling | 3.6 | ARPC1B, FGF16, ITGB4, MET, VEGFA |
| Virus Entry via Endocytic Pathways | 3.5 | ITGB4, PRKCE, RAP1A, RAP1B |
| UVC-Induced MAPK Signaling | 3.37 | PRKCE, RAP1A, RAP1B |
| fMLP Signaling in Neutrophils | 3.36 | ARPC1B, PRKCE, RAP1A, RAP1B |
| Superpathway of Inositol Phosphate Compounds | 3.35 | ATP1A1, CDC25B, INPPL1, PTEN, SET |
| IL-6 Signaling | 3.34 | RAP1A, RAP1B, SHC1, VEGFA |
| Estrogen Receptor Signaling | 3.19 | MED1, RAP1A, RAP1B, SHC1 |
| White Adipose Tissue Browning Pathway | 3.12 | CREB1, FGFR3, SIRT1, VEGFA |
| IL-2 Signaling | 3.1 | RAP1A, RAP1B, SHC1 |
| D-myo-inositol (1, 4, 5, 6)-Tetrakisphosphate Biosynthesis | 3.07 | ATP1A1, CDC25B, PTEN, SET |
| D-myo-inositol (3, 4, 5, 6)-tetrakisphosphate Biosynthesis | 3.07 | ATP1A1, CDC25B, PTEN, SET |
| Role of JAK1 and JAK3 in γc Cytokine Signaling | 2.99 | RAP1A, RAP1B, SHC1 |
| D-myo-inositol-5-phosphate Metabolism | 2.92 | ATP1A1, CDC25B, PTEN, SET |
| Pyridoxal 5'-phosphate Salvage Pathway | 2.88 | DYRK1A, PLK1, PRKCE |
| FcγRIIB Signaling in B Lymphocytes | 2.82 | RAP1A, RAP1B, SHC1 |
| IL-7 Signaling Pathway | 2.8 | BCL6, MET, SHC1 |
| 3-phosphoinositide Biosynthesis | 2.77 | ATP1A1, CDC25B, PTEN, SET |
| PEDF Signaling | 2.77 | RAP1A, RAP1B, ZEB1 |
| NF-κB Activation by Viruses | 2.75 | PRKCE, RAP1A, RAP1B |
| D-myo-inositol (1, 3, 4)-trisphosphate Biosynthesis | 2.67 | INPPL1, PTEN |
| Regulation of IL-2 Expression in Activated and Anergic T Lymphocytes | 2.64 | RAP1A, RAP1B, VAV2 |
| Th17 Activation Pathway | 2.61 | HIF1A, MTOR, RUNX1 |
| UVA-Induced MAPK Signaling | 2.54 | MTOR, RAP1A, RAP1B |
| Mouse Embryonic Stem Cell Pluripotency | 2.49 | RAP1A, RAP1B, SMAD1 |
| Paxillin Signaling | 2.43 | ITGB4, RAP1A, RAP1B |
| Superpathway of D-myo-inositol (1, 4, 5)-trisphosphate Metabolism | 2.42 | INPPL1, PTEN |
| Sumoylation Pathway | 2.42 | ETS1, SIRT1, ZEB1 |
| T Cell Receptor Signaling | 2.41 | RAP1A, RAP1B, VAV2 |
| Salvage Pathways of Pyrimidine Ribonucleotides | 2.37 | DYRK1A, PLK1, PRKCE |
| GP6 Signaling Pathway | 2.26 | LAMC2, PRKCE, RAP1B |
| Inhibition of Matrix Metalloproteases | 2.23 | MMP3, MMP9 |
| CCR3 Signaling in Eosinophils | 2.22 | PRKCE, RAP1A, RAP1B |
| IL-23 Signaling Pathway | 2.15 | HIF1A, RUNX1 |
| Human Embryonic Stem Cell Pluripotency | 2.12 | BMP1, FGFR3, SMAD1 |
| UVB-Induced MAPK Signaling | 2.01 | MTOR, PRKCE |
| PKCθ Signaling in T Lymphocytes | 1.97 | RAP1A, RAP1B, VAV2 |
| Role of CHK Proteins in Cell Cycle Checkpoint Control | 1.93 | E2F1, PLK1 |
| EGF Signaling | 1.9 | MTOR, SHC1 |
| CXCR4 Signaling | 1.86 | PRKCE, RAP1A, RAP1B |
| Autophagy | 1.82 | CTSC, MTOR |
| Mitotic Roles of Polo-Like Kinase | 1.81 | CDC25B, PLK1 |
| Regulation of Cellular Mechanics by Calpain Protease | 1.81 | RAP1A, RAP1B |
| Remodeling of Epithelial Adherens Junctions | 1.79 | ARPC1B, MET |
| Sertoli Cell-Sertoli Cell Junction Signaling | 1.74 | PTEN, RAP1A, RAP1B |
| Agranulocyte Adhesion and Diapedesis | 1.74 | FN1, MMP3, MMP9 |
| Production of Nitric Oxide and Reactive Oxygen Species in Macrophages | 1.74 | PRKCE, RAP1A, RAP1B |
| Growth Hormone Signaling | 1.71 | IGF1R, PRKCE |
| Antiproliferative Role of Somatostatin Receptor 2 | 1.64 | RAP1A, RAP1B |
| Gap Junction Signaling | 1.64 | PRKCE, RAP1A, RAP1B |
| Chemokine Signaling | 1.62 | RAP1A, RAP1B |
| HIPPO signaling | 1.6 | CD44, SMAD1 |
| ATM Signaling | 1.5 | CREB1, ZEB1 |
| Calcium Transport I | 1.4 | ATP2B1 |
| iCOS-iCOSL Signaling in T Helper Cells | 1.36 | PTEN, SHC1 |
| Prostanoid Biosynthesis | 1.35 | PTGS2 |
| Phagosome Formation | 1.3 | FN1, PRKCE |
| Granzyme A Signaling | 1.27 | SET |
| Androgen Signaling | 1.22 | PRKCE, SHC1 |
| D-myo-inositol (1, 4, 5)-trisphosphate Degradation | 1.21 | INPPL1 |
| Fatty Acid α-oxidation | 1.19 | PTGS2 |
| Estrogen-mediated S-phase Entry | 1.14 | E2F1 |
| 1D-myo-inositol Hexakisphosphate Biosynthesis II (Mammalian) | 1.14 | INPPL1 |
| Role of Pattern Recognition Receptors in Recognition of Bacteria and Viruses | 1.14 | CREB1, PRKCE |
| Phagosome Maturation | 1.12 | CTSC, PRDX6 |
| HMGB1 Signaling | 1.09 | RAP1A, RAP1B |
| Cdc42 Signaling | 1.07 | ARPC1B, VAV2 |
| Glutathione Redox Reactions I | 1.07 | PRDX6 |
| Germ Cell-Sertoli Cell Junction Signaling | 1.05 | RAP1A, RAP1B |
| Role of JAK2 in Hormone-like Cytokine Signaling | 1.03 | SHC1 |
| Granulocyte Adhesion and Diapedesis | 1.02 | MMP3, MMP9 |
| MIF-mediated Glucocorticoid Regulation | 1.01 | PTGS2 |
| Cell Cycle Regulation by BTG Family Proteins | 0.996 | E2F1 |
| Mechanisms of Viral Exit from Host Cells | 0.955 | PRKCE |
| MIF Regulation of Innate Immunity | 0.917 | PTGS2 |
| Triacylglycerol Degradation | 0.851 | PRDX6 |
| Transcriptional Regulatory Network in Embryonic Stem Cells | 0.842 | SET |
| CD40 Signaling | 0.77 | PTGS2 |
| IL-17A Signaling in Airway Cells | 0.762 | PTEN |
| T Helper Cell Differentiation | 0.717 | BCL6 |
| Caveolar-mediated Endocytosis Signaling | 0.712 | ITGB4 |
| Heparan Sulfate Biosynthesis (Late Stages) | 0.688 | PRDX6 |
| Cyclins and Cell Cycle Regulation | 0.682 | E2F1 |
| Heparan Sulfate Biosynthesis | 0.638 | PRDX6 |
| CCR5 Signaling in Macrophages | 0.606 | PRKCE |
| CD28 Signaling in T Helper Cells | 0.52 | ARPC1B |
| IL-12 Signaling and Production in Macrophages | 0.489 | PRKCE |
| Dendritic Cell Maturation | 0.386 | CREB1 |

The enriched pathways were based on the right-tailed Fisher’s exact test (adjusted for False Discover Rate at 5%) that are graphed as negative log p value. These pathways indicate the likelihood of an association of genes to the pathway in MF versus HM fed piglets at different time points.
